# Machine Learning Meta-analysis of Large Metagenomic Datasets: Tools and Biological Insights

**DOI:** 10.1371/journal.pcbi.1004977

**Published:** 2016-07-11

**Authors:** Edoardo Pasolli, Duy Tin Truong, Faizan Malik, Levi Waldron, Nicola Segata

**Affiliations:** 1 Centre for Integrative Biology, University of Trento, Trento, Italy; 2 Graduate School of Public Health and Health Policy, City University of New York, New York, New York, United States of America; University of California Davis, UNITED STATES

## Abstract

Shotgun metagenomic analysis of the human associated microbiome provides a rich set of microbial features for prediction and biomarker discovery in the context of human diseases and health conditions. However, the use of such high-resolution microbial features presents new challenges, and validated computational tools for learning tasks are lacking. Moreover, classification rules have scarcely been validated in independent studies, posing questions about the generality and generalization of disease-predictive models across cohorts. In this paper, we comprehensively assess approaches to metagenomics-based prediction tasks and for quantitative assessment of the strength of potential microbiome-phenotype associations. We develop a computational framework for prediction tasks using quantitative microbiome profiles, including species-level relative abundances and presence of strain-specific markers. A comprehensive meta-analysis, with particular emphasis on generalization across cohorts, was performed in a collection of 2424 publicly available metagenomic samples from eight large-scale studies. Cross-validation revealed good disease-prediction capabilities, which were in general improved by feature selection and use of strain-specific markers instead of species-level taxonomic abundance. In cross-study analysis, models transferred between studies were in some cases less accurate than models tested by within-study cross-validation. Interestingly, the addition of healthy (control) samples from other studies to training sets improved disease prediction capabilities. Some microbial species (most notably *Streptococcus anginosus*) seem to characterize general dysbiotic states of the microbiome rather than connections with a specific disease. Our results in modelling features of the “healthy” microbiome can be considered a first step toward defining general microbial dysbiosis. The software framework, microbiome profiles, and metadata for thousands of samples are publicly available at http://segatalab.cibio.unitn.it/tools/metaml.

## Author Summary

The human microbiome–the entire set of microbial organisms associated with the human host–interacts closely with host immune and metabolic functions and is crucial for human health. Significant advances in the characterization of the microbiome associated with healthy and diseased individuals have been obtained through next-generation DNA sequencing technologies, which permit accurate estimation of microbial communities directly from uncultured human-associated samples (e.g., stool). In particular, shotgun metagenomics provide data at unprecedented species- and strain- levels of resolution. Several large-scale metagenomic disease-associated datasets are also becoming available, and disease-predictive models built on metagenomic signatures have been proposed. However, the generalization of resulting prediction models on different cohorts and diseases has not been validated. In this paper, we comprehensively assess approaches to metagenomics-based prediction tasks and for quantitative assessment of microbiome-phenotype associations. We consider 2424 samples from eight studies and six different diseases to assess the independent prediction accuracy of models built on shotgun metagenomic data and to compare strategies for practical use of the microbiome as a prediction tool.

## Introduction

The human microbiome constitutes the whole set of microbial organisms associated with the human host. It has been shown to be crucial for human health and for the development and maintenance of the immune system and for several metabolic activities [1–3]. Significant effort has been devoted to its characterization in healthy individuals and subjects with a variety of diseases such as inflammatory bowel diseases [4,5], obesity [6,7], and type-2 diabetes [8]. Consequently, the potential use of the microbiome as a diagnostic tool is a promising line of investigation [9]. In addition, even when the findings are not immediately relevant for the clinical setting, identifying associations between the microbiome and specific diseases is essential for follow-up mechanistic studies.

Next-generation DNA sequencing technologies permit comprehensive profiling of the microbial communities from human-associated samples, and have now been sufficiently widely employed to enable meta-analysis for discovering patterns common to independent studies. Meta-analysis has been broadly adopted in other genomics applications, such as for analysis of microarray or RNA-seq data, where multiple studies have been performed for a similar purpose including identifying gene expression signatures of specific human cancers. The general objectives of meta-analysis include proposing new classifiers [10], comparing different classification methods [11], finding a common transcriptional profile [12], and evaluating generalization of prediction models across different studies [13]. In genomics, rigorous meta-analyses are crucial both to validate the findings of each single study, and for providing robust models for clinical purposes.

The most common and cost-effective approach for microbiome characterization to date targets the 16S rRNA gene as taxonomic marker [14]. Meta-analyses and independent validation of such experimental approach have identified differences in microbiome composition or function by body site, age, and disease state [15–17], and have been conducted to determine the most effective techniques for disease classification [18]. More recently, shotgun metagenomics [19] provided expanded resolution to the level of microbial species [20–22] and strain [23], to the fungal and viral kingdoms [24], and to the level of individual genes across the metagenome [25,26]. The decreasing cost of shotgun metagenomics is rapidly increasing the number of available human disease-associated datasets; however, the generalization of resulting prediction models is still unclear.

Improved resolution and lower variability of shotgun metagenomics hold the promise to provide improved generalization of microbial signatures over 16S rRNA sequencing [27]. Meta-analyses on specific host characteristics have been performed (e.g., with respect to host age [28]). The importance of cross-cohort consistency and validation of predictions has also been recognized, with some works assessing the structure of the microbiome in European cohorts [29] and combined European-American cohorts [21, 30]. Some studies focusing on the link between host conditions and microbiome further provided a validation step with respect to other single investigations [31, 32]. Although these works provided a first assessment on the transferability of condition-associated microbiome features across cohorts, no systematic assessments have been performed on clinical outcomes using the full archive of shotgun metagenomic data now publicly available, and no convenient software frameworks for doing so are available in the community.

In this study we uniformly process 2424 shotgun metagenomic samples from eight studies to assess the independent prediction accuracy of models built on metagenomic data and to compare strategies for practical use of the microbiome as a prediction tool. The software framework and the microbiome profiles for thousands of samples are made publicly available.

## Results and Discussion

**Fig 1:**
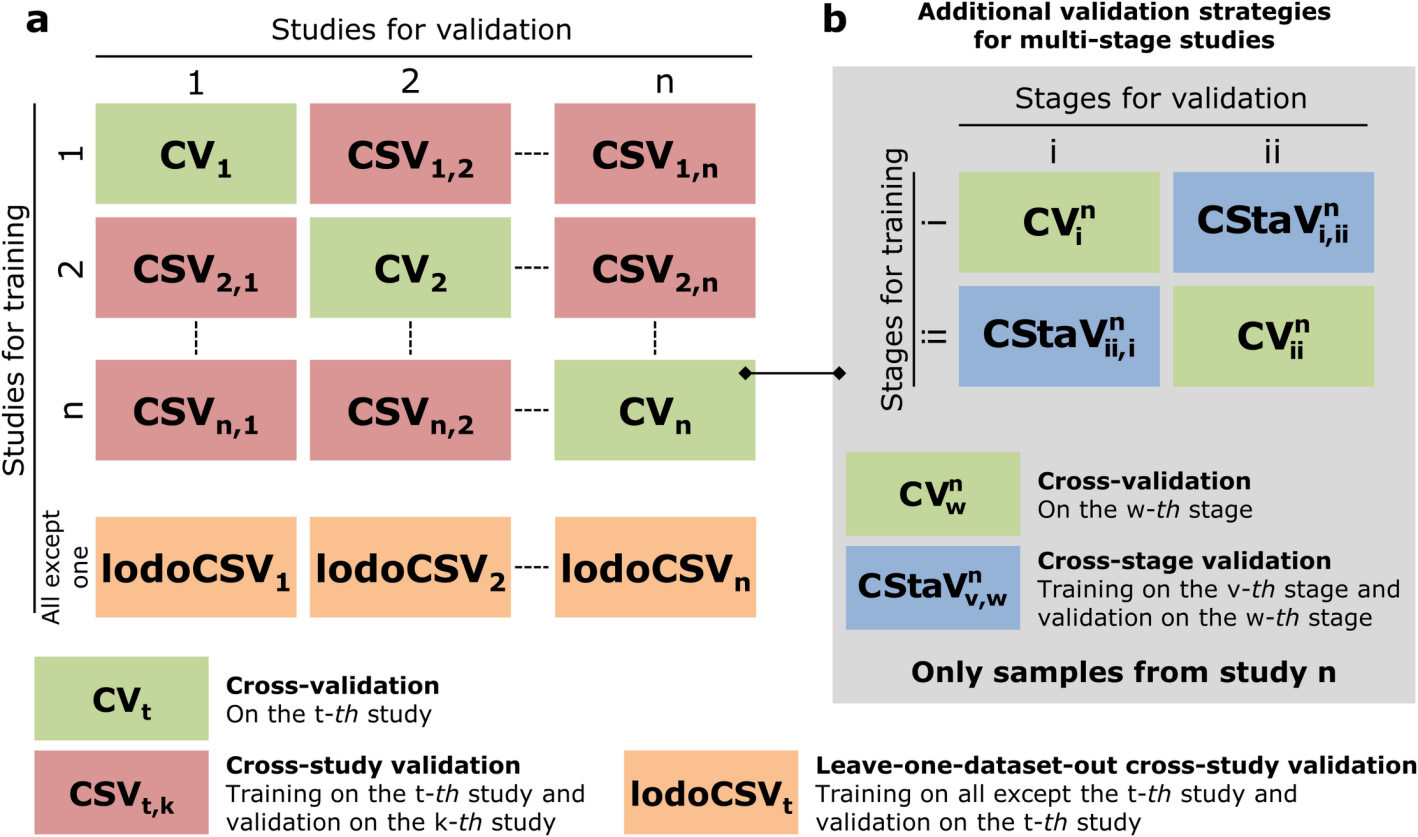
Validation strategies implemented in the developed framework. (**a**) Main strategies include cross-validation on single studies and cross-validation across multiple studies. (**b**) Additional strategies when multiple stages are available from the same study.

We evaluated alternative approaches to metagenomics-based prediction tasks, and assessed the strength of microbiome-phenotype associations using publicly available raw sequence data. For this purpose, we developed a machine-learning software framework which uses as features quantitative microbiome profiles, including species-level relative abundances and presence of species- and strain-specific markers (see **Methods**). Our multi-level validation strategy includes the assessment of microbiome models on single cohorts, across stages of the same study, across different studies, and across target outcomes and conditions (**Fig 1**). The software and validation framework is publicly available at http://segatalab.cibio.unitn.it/tools/metaml and was applied on a total of 2424 publicly available metagenomic samples from eight large-scale studies (see **Table 1** and **Methods**). All samples were processed with MetaPhlAn2 [21] for quantitative species- and subspecies-level taxonomic profiling after standard sequencing data pre-processing (see **Methods**).

**Table 1:** Summary of the datasets considered in the experiments.

Dataset name	Body site	Disease	#stages	#case samples	#control samples	Average reads per sample (std)	Reference
Cirrhosis	Gut	Liver Cirrhosis	2	118	114	51.6M (30.9M)	[33]
Colorectal	Gut	Colorectal Cancer	1	48	73	60.0M (25.5M)	[34]
HMP	Several	None	1	-	981	61.1M (51.2M)	[1]
IBD	Gut	Inflammatory Bowel Diseases	1	25	85	45.2M (18.4M)	[35]
Obesity	Gut	Obesity	1	164	89	68.2M (23.2M)	[31]
Skin	Skin	None	1	-	287	24.7M (38.1M)	[36]
T2D	Gut	Type 2 diabetes	2	170	174	40.2M (11.8M)	[37]
WT2D	Gut	Type 2 diabetes	1	53	43	31.0M (17.6M)	[32]

### Cross-validation studies revealed good capabilities for disease prediction

We first assessed the prediction power of metagenomic data in linking the gut microbiome with disease states. For such purpose, we considered six available disease-associated metagenomic datasets spanning five diseases: liver cirrhosis [33], colorectal cancer [34], inflammatory bowel diseases (IBD) [35], obesity [31], and type 2 diabetes (two distinct studies—[37] and [32]). Each dataset was analyzed independently using cross-validation (denoted as CV in **Fig 1**), which repeatedly uses part of the samples with associated known phenotype for learning the statistical model, and the remainder for validating the predictions (see **Methods**). The support vector machines (SVM) [38] and random forest (RF) [39] classifiers were used for this evaluation as they are state-of-the-art approaches and are appropriate for this type of data [18]. We also evaluated Lasso [40] and elastic net (ENet) [41] regularized multiple logistic regression. Neural networks [42] and Bayesian logistic regression [43] represent other possible alternatives not evaluated here.

**Fig 2:**
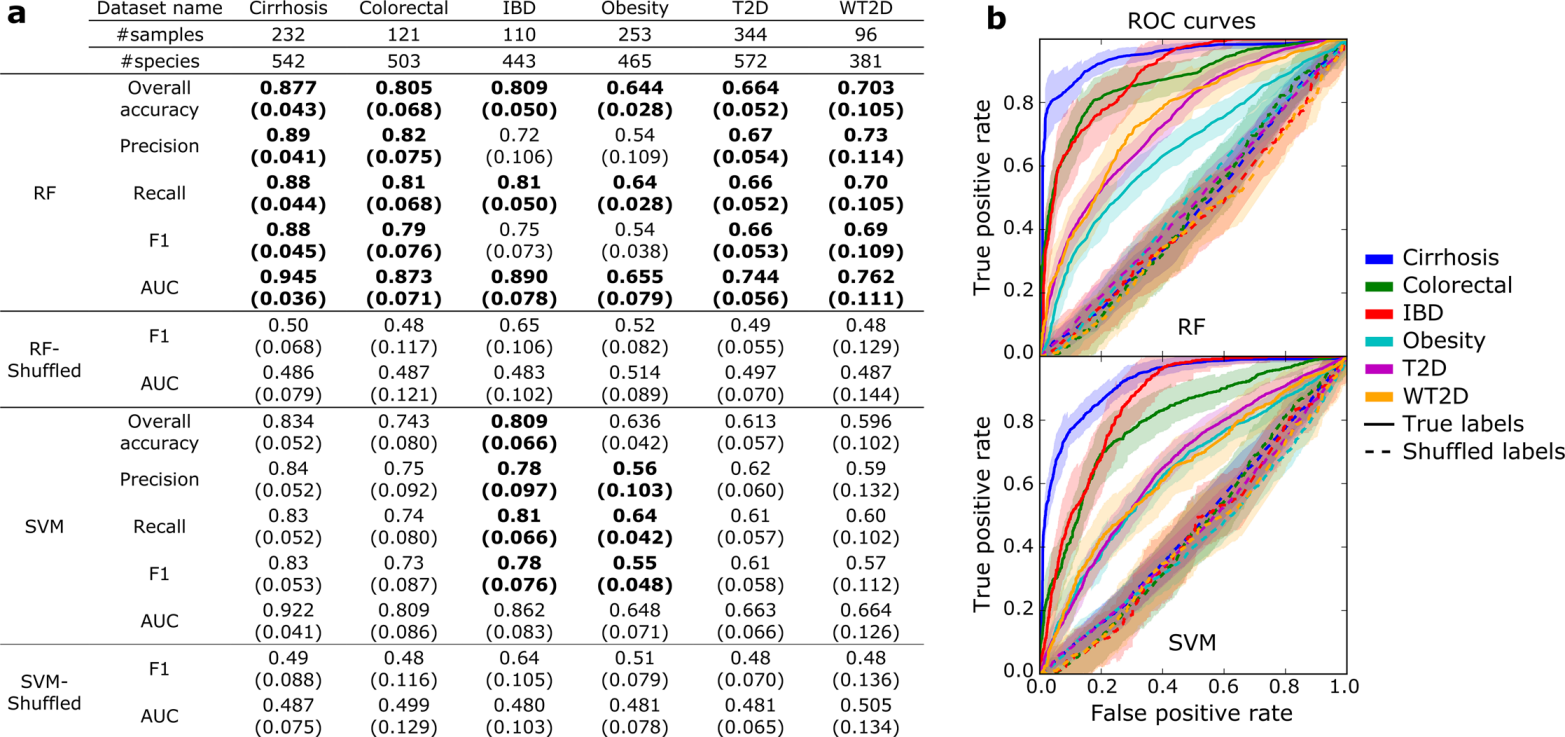
Cross-validation analysis for disease discrimination on six different datasets. Species abundance was used as microbiome feature. (**a**) Prediction performance metrics for different diseases versus healthy controls. The margin of errors are reported in parenthesis. In bold we report the best value for each dataset. (**b**) Average ROC curves (over folds) with confidence intervals for random forests (RF) and support vector machines (SVM).

Prediction performance was evaluated by the area under the curve (AUC) metric, which summarizes true positive and false positive rates and is robust to unequal proportions of each outcome. Using MetaPhlAn2 species abundance [21] as input data produced high accuracy for disease classification (**Fig 2**), although prediction performance varied considerably between datasets. The most predictable disease state appears to be liver cirrhosis (AUC = 0.945, 95% CI: 0.909–0.981 for the best classifier), followed by colorectal cancer (AUC = 0.873, 95% CI: 0.802–0.944), and IBD (AUC = 0.890, 95% CI: 0.812–0.968). For IBD we considered Crohn and ulcerative colitis patients together due to the low number of cases in the datasets compared to controls (as general rule at least ten samples per class are required for reliable prediction models). Stronger signatures might be found when considering the two conditions separately with adequate sample size, as it has been observed that some bacterial features are specific to Crohn disease only [16]. Confounding factors such as active treatment could of course lead to overestimated prediction capabilities [44, 45], but we adopted here the same contrasting approach used in the original works.

For the other diseases we achieved lower discrimination capabilities, suggesting less dramatic microbial shifts in the patients. For type 2 diabetes, although the two considered datasets have independently sampled and geographically distinct cohorts, we obtained very similar AUC values for both (0.744, 95% CI: 0.688–0.800 and 0.762, 95% CI: 0.651–0.873 for T2D and WT2D, respectively). Prediction of obesity generated the lowest AUC (0.655, 95% CI: 0.576–0.734). Despite a wide range of classification performances, all investigated datasets showed a substantial level of association between disease and the microbiome (**Fig 2**), with AUC values significantly higher than those obtained by the same classifier applied to the same data with shuffled class labels (*p*-values ranging from 9.9 × 10^−3^ for obesity to 5.6 × 10^−7^ for cirrhosis, **S1 Table**).

Comparing the accuracy of SVM and RF classifiers, RFs exhibited in all cases similar or better results than SVM. In particular, accuracies differed substantially for three datasets: AUC increased from 0.809 to 0.873 for colorectal, from 0.663 to 0.744 for T2D (difference also supported by statistical significance, *p-*value 0.011, see **S1 Fig**), and from 0.664 to 0.762 for WT2D. In two cases, slight improvements were verified: AUC increased from 0.922 to 0.945 for cirrhosis and from 0.862 to 0.890 for IBD. Methodologically, our results thus suggested the use of RFs for disease prediction from species abundances.

### Feature selection and strain-specific markers improve prediction accuracy

**Fig 3:**
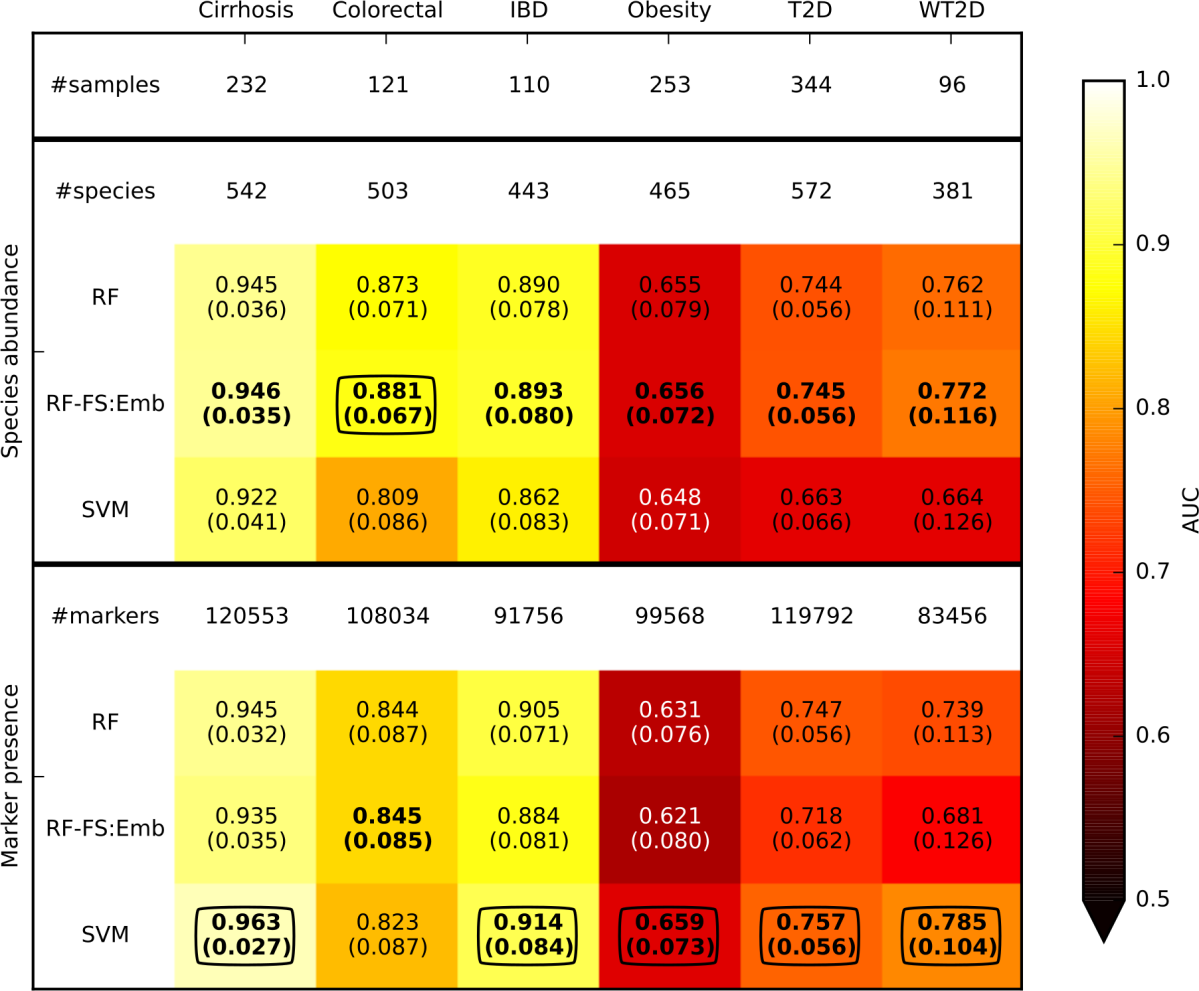
Prediction performances (assessed using AUC) for disease discrimination in different cross-validation studies. Species abundance and marker presence are the microbiome features used by the classifiers. The best value for each dataset and feature type (i.e., species abundance or marker presence) are in bold, and the overall best values for each dataset are circled. RF and SVM are applied on the entire set of features whereas RF-FS:Emb incorporates a feature selection step (see **Methods**). Margins of error are reported in parenthesis.

We then investigated how feature selection, i.e., the procedure of selecting a reduced subset of relevant discriminative features, impacts the prediction accuracy. To this end, we used the RF classifier that implicitly embeds a feature selection step during the model generation phase (see **Methods**). Feature selection produced a slight improvement of the AUC in all the cases when the model was generated on a reduced set of species (**Fig 3**). The advantage of this procedure is twofold. In addition to the increased accuracy, it enables biomarker discovery by detecting the (few) species that are most useful to discriminate between “healthy” and “diseased” subjects. These most discriminative species may be prioritized when performing follow-up and validation analyses, and the reduced complexity of the model potentially enables additional evaluations on low-throughput assays. However, the best accuracies were obtained with still relatively high numbers of species, i.e., more than 60 (**S2 Fig**). This confirms the complexity of microbial ecosystems where the combination of few species is probably not sufficient to characterize the microbiome associated with complex diseases.

We then investigated the use of strain-specific markers, as opposed to species-level taxonomic abundance, by applying the same classification and cross-validation methods to strain-specific microbial features generated by MetaPhlAn2. Given their strain-specificity, adopting markers as features let us also test the hypothesis that complex diseases are associated with the presence of specific strains or subspecies rather than only species-level abundances. Consistent with this hypothesis, better predictions were obtained from markers (**Fig 2**) than species abundance, with differences that were statistically significant for one dataset (**S1 Fig**). This was obtained using SVM with linear kernel, which in this context is more practical to use than RF and SVM with more complex kernels due to the very high dimensionality (~100K features) of the data. Focusing on SVM, markers gave statistically significant improvements with respect to species abundances in half of the datasets (**S1 Fig**). Moreover, RF in combination with feature selection (RF-FS:Emb) achieved satisfactory classification results, i.e., average accuracies were usually worse than SVM but with no statistically significant difference (**S1 Fig**) even using a very limited portion (<0.2%, **S2 Fig**) of the investigated markers. The biomarker discovery step here is of particular interest because it permits identification of a limited set of strain-specific markers potentially directly involved in the association with disease.

We also considered alternative approaches to feature selection based on Lasso and ENet (see **Methods**). Applying Lasso or ENet as pure classifiers, which implicitly incorporates the feature selection and classification steps, did not give satisfactory results, with AUC worse than RF or SVM for both species abundance and marker features (**S3 Fig**). Better accuracies were obtained by using them for feature selection only, followed by RF or SVM classification. However, both Lasso and ENet feature selection in general worsened the performance of RF and SVM without prior feature selection. Finally, ENet worked better than Lasso, although it was associated with more time-consuming tuning of its free parameters on a two-dimensional grid.

### Detection of the disease-associated microbial features

**Fig 4:**
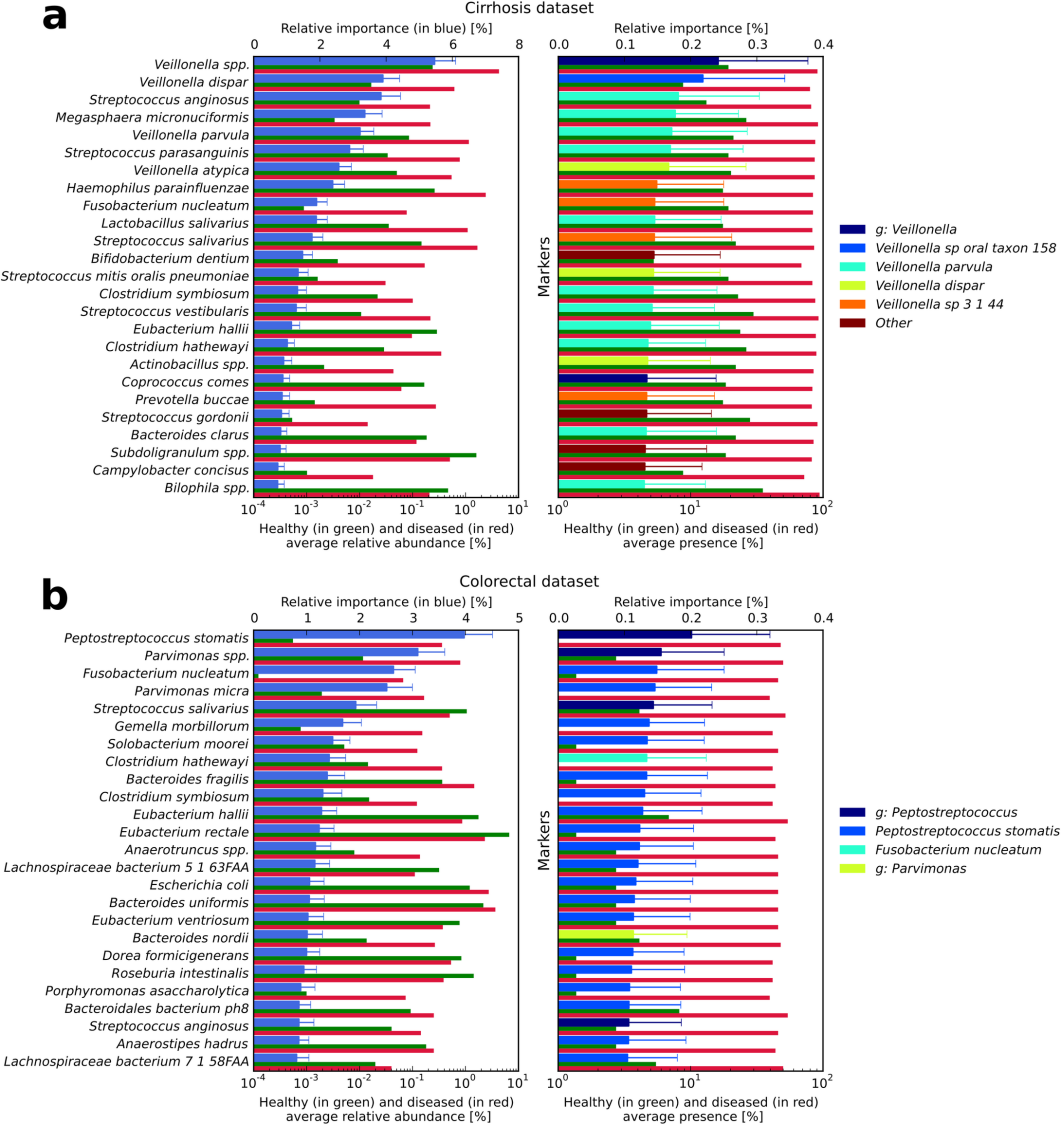
**Most important discriminating species (left) and markers (right) identified by RF for disease discrimination in the (a) cirrhosis and (b) colorectal cancer cross-validation studies.** In the left panels, for each species reported on the vertical axis, the top bar (in blue) corresponds to the feature relative importance (with standard deviation reported with error bars) and the two bottom bars refer to the average relative abundance for healthy (in green) and diseased (in red) samples. In the right panels, for each marker the top bar is coloured according to the corresponding species and the two bottom bars refer to the average marker presence.

Feature selection can also be used for biomarker discovery, and several tools have been developed specifically for this task in metagenomics [46–48]. The approach proposed here (RF with embedded feature selection) focuses on the set of features with the most discriminating power rather than on strictly statistical assessments [46] or statistical assessment coupled with effect size [47]. The implemented tool automatically plots the most relevant species (or markers) with the importance factor (see **Methods**) along with the average relative abundance (or average presence) associated with the different considered classes. We observed a reasonable level of overlap between the detected species and markers, as the most discriminative markers tended to represent strains of the most discriminative species. Interestingly, for all the considered datasets (**Fig 4** and **S4 Fig**) the importance factor attributed to each species (or marker) was not well correlated with its average relative abundance (or presence) in the samples (maximum correlation of 0.49 for the T2D dataset, **S5 Fig**). In several cases, we detected relevant species with partial prevalence but highly discriminative potential between “healthy” and “diseased” subjects. For example, *Peptostreptococcus stomatis* resulted the most discriminative species in the colorectal dataset with an average relative abundance in the samples less than 0.15%.

In the cirrhosis dataset, the most relevant taxonomic abundances were enriched in diseased patients. The top features were especially related to the *Veillonella* (*Veillonella spp*., *Veillonella dispar*, *Veillonella parvula*, and *Veillonella atypica*) and *Streptococcus* genera (*Streptococcus anginosus* and *Streptococcus parasanguinis*) in addition to *Haemophilus parainfluenzae*, which is consistent with findings of the original study [33]. Species belonging to *Veillonella* and *Streptococcus* are typical colonizers of the oral cavity, but they are often overgrown in the small intestine in patients affected by liver cirrhosis, thus suggesting the invasion of the gut from the mouth in these patients [33]. Moreover, species such as *Veillonella spp*., *V*. *dispar*, *V*. *atypica*, and *S*. *anginosus* were already associated with opportunistic infections [33]. Also the *H*. *parainfluenzae* pathogen may arrive to the gut from the oral cavity [33]. In the colorectal dataset we identified five major species: *P*. *stomatis*, *Fusobacterium nucleatum* (both enriched in diseased patients) and *Streptococcus salivarius* (depleted in diseased subjects) as found in the original study [34], in addition to *Parvimonas spp*. and *Parvimonas micra*.

We then compared the discriminative species across datasets through hierarchical clustering (**S6 Fig**). We found some species that were distinctive of one disease only as it is the case for *P*. *stomatis*, *P*. *micra* and *Gemella morbillorum* in colorectal cancer, multiple *Veillonella* species in cirrhosis, and, partially, *Bifidobacterium bifidum* and *Lachnospiraceae* in IBD. Interestingly, *F*. *nucleatum* was highly discriminant both in colorectal cancer and cirrhosis, suggesting the presence of a similar dysbiosis niche for this organism. Overall, the discriminative species for the two diabetes datasets and the obesity dataset had lower weights, consistent with the lower classification performances achieved with them. Moreover, the pattern of discriminative species for these two datasets clustered together (**S6 Fig**), suggesting similar dysbiotic configurations of the gut microbiome for obesity and type-2 diabetes. Some species were also found in the set of top discriminative features for all the studies, in particular *S*. *salivarius*, *S*. *anginosus*, *V*. *parvula*, *Roseburia intestinalis*, and *Coprococcus comes*. These species might thus be biomarkers of general dysbiosis or ecological community stress in non-healthy states, and should be recognized as such in future disease-microbiome association studies.

### Extension to non-disease classification problems

We extended the cross-validation analysis by evaluating the predictability for non-disease based classification problems. Gender discrimination (**S7 Fig, part a**) exhibited in general low classification accuracy with an AUC close or less than 0.6 for most of the considered datasets. However, statistically significant discrimination was verified in some cases (AUC equal to 0.662 and 0.796 for skin and IBD dataset, respectively, both p < 0.05 by permutation test with shuffled labels), which may suggest some gender-dependent differences in the human microbiome as highlighted by recent studies [49]. High classification accuracy in body site prediction in the Human Microbiome Project (HMP) dataset (AUC = 0.96), is consistent with previously reported large differences in the microbiome composition among different body areas [1], and provided validation of the proposed tool for multi-class classification problems (**S8 Fig**). The confusion matrix revealed moderate misclassification between nasal and skin body sites, which may be due to nasal samples being taken from the anterior nares (external part of the nostrils), and thus having relatively similar biochemical characteristics compared to skin samples from the retroauricular crease.

### Metagenomic disease-predictive models show strong cross-stage generalization

The cross-validation studies discussed in previous sections permitted evaluation of the predictability of different disease states from the human microbiome. However, they are not necessarily a good proxy to evaluate the generalization of the prediction model to independent validation samples, a scenario more relevant to a clinical setting but that has been scarcely investigated. Specifically, how do prediction models perform when applied to samples generated in an independent clinical and laboratory study? We address this question for several problems of increasing complexity (denoted as CStaV in **Fig 1**).

**Fig 5:**
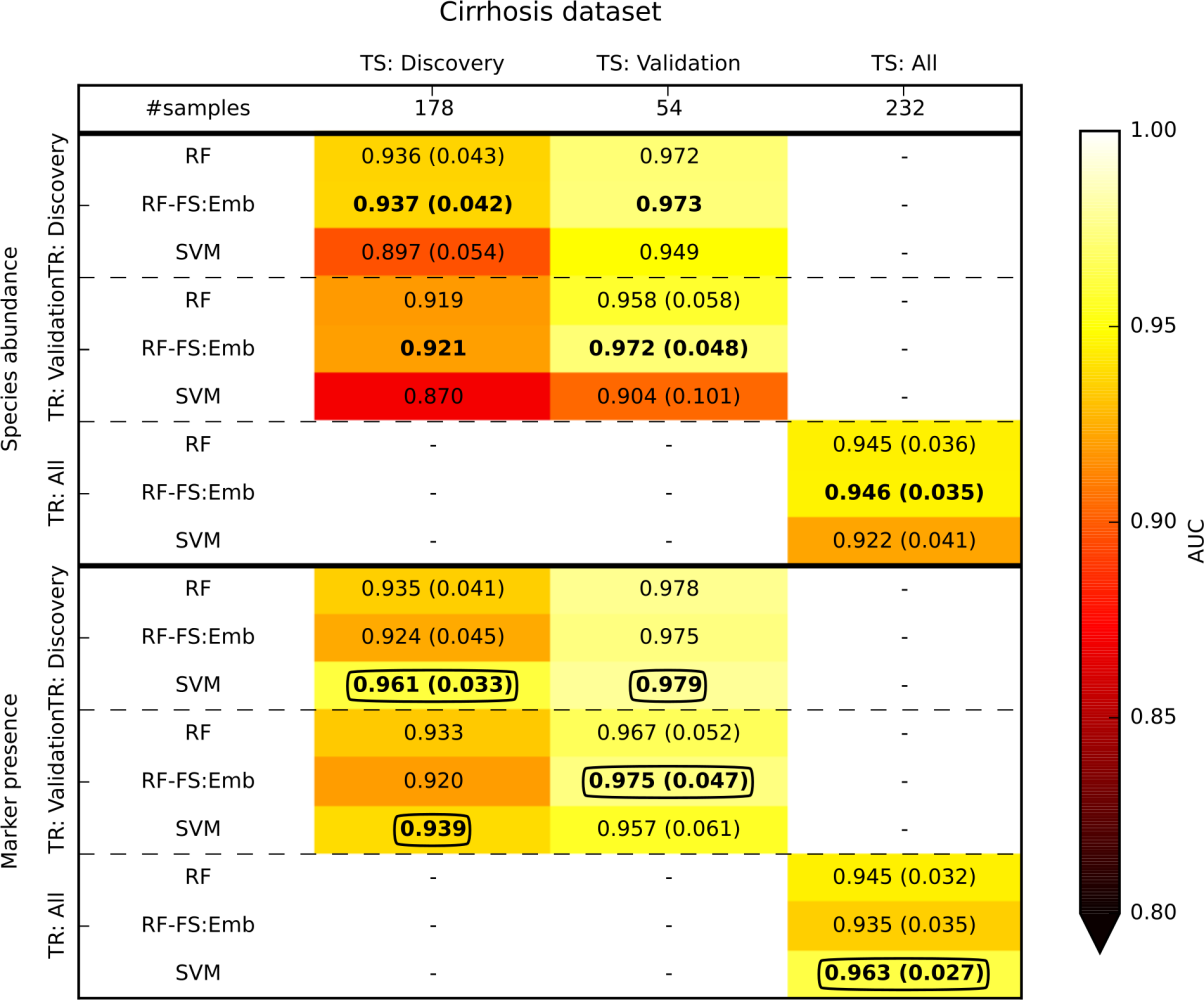
Cross-stage analysis of disease discrimination in the cirrhosis dataset, which was generated in two independent stages (discovery and validation). The “All” columns and rows show results when all samples are combined. When the training (TR) and test (TS) stages coincide, the analysis was done in cross-validation (with the margin of error reported in parenthesis). In the other cases, the model was generated on TR and then applied to TS. In bold we report the best value for each scenario and feature type (i.e., species abundance or marker presence), and circled are the overall best value for each scenario.

We first considered the cirrhosis dataset, in which the samples were acquired in two distinct stages named “discovery” and “validation” (**Fig 5**). The generalization of the model was evaluated by (i) generating the model on the samples of the training (TR) stage and (ii) applying it on the test (TS) stage. For comparison, we also report the cross-validation results obtained on each specific stage. In general, we found that the model was transferred properly from one stage to the other. In fact, RF applied on species abundance produced an AUC value on the discovery stage that was only slightly decreased from 0.936 (for cross-validation) to 0.919. For the validation stage we actually obtained an increase from 0.958 (for cross-validation) to 0.972, and the marker-based predictions achieved slightly better but overall consistent values (**Fig 5**). Finally, we note that the AUC achieved on each specific stage were in line with the AUC exhibited by cross-validation using the entire set of samples (0.945).

**Fig 6:**
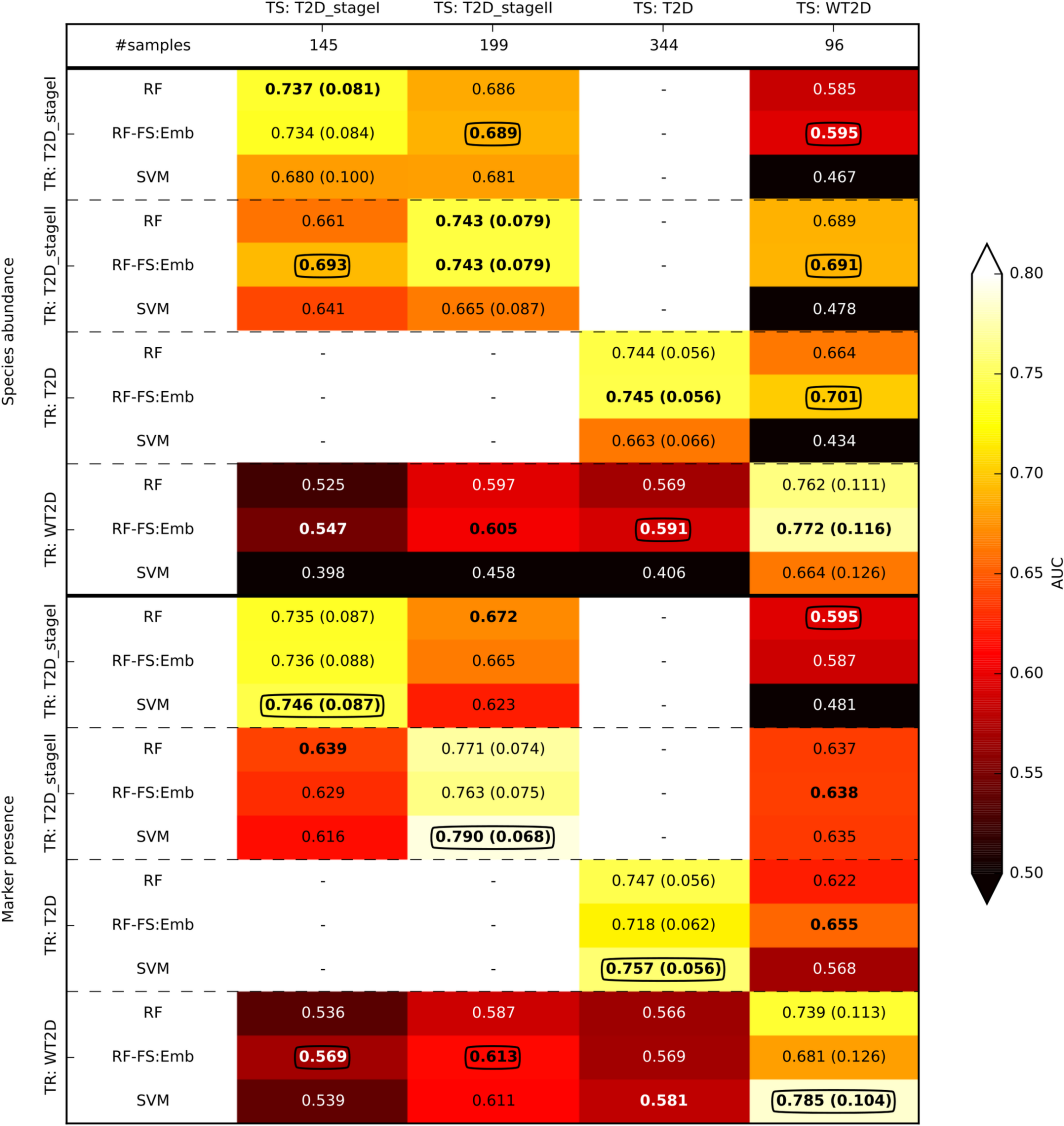
AUC by cross-stage and cross-study analysis for T2D discrimination in the T2D and WT2D datasets. When the training (TR) and test (TS) sets coincide, the analysis was done in cross-validation (with the margin of error reported in parenthesis). In the other cases, the model was generated on TR and then applied to TS. In bold we report the best value for each setting and feature type (i.e., species abundance or marker presence), and circled are the overall best value for each scenario.

A similar analysis was done on the T2D dataset, in which samples were collected in two different stages (stageI and stageII, **Fig 6**). We verified sufficient generalization of the model across the two stages, although we observed a decrease in accuracy relative to cross-validation. AUC for RF on species abundance decreased from a cross-validation value of 0.737 (0.735 for marker presence) to 0.661 (0.639) for stageI, and from 0.743 (0.771) to 0.686 (0.672) for stageII. In general, the results obtained on the cirrhosis and T2D datasets provide reasonably good generalization of the model when applied across disease stages, i.e., to independent samples/batches from the same study. This implies that the samples, although associated with different subjects and acquired at different time points, share common characteristics such the population of study, sample collection approach, DNA extraction protocol, sequencing technology, and analysis strategy [19].

### Cross-study generalization is improved by including healthy samples from other cohorts

Cross-study validation (denoted as CSV in **Fig 1**) is a more difficult standard of validation than cross-stage validation, in that training and validation are performed in completely independent studies targeting the same disease. We focused on type-2 diabetes, for which two distinct datasets are available (i.e., T2D and WT2D). The two datasets presented very different population characteristics as T2D targeted Chinese subjects while WT2D enrolled European women. Still, we observed generalization from one study to the other, (**Fig 6**), although cohort effects clearly affected the results. For validation on the T2D dataset, the AUC for RF on species abundance decreased from a cross-validation value of 0.744 (0.747 for marker presence) to 0.569 (0.566) when the model was constructed on the WT2D dataset. Similarly, for validation on the WT2D dataset, AUC decreased from a cross-validation value of 0.762 (0.739) to 0.664 (0.622). Different results were achieved by transferring the model to WT2D from the two different experimental stages of the T2D dataset. We obtained an AUC of 0.585 (0.595) and 0.689 (0.637) by transferring the model from T2D_stageI and T2D_stageII, respectively, indicating that T2D_stageII was more similar than T2D_stageI to WT2D. This similarity was consistent with integrative correlation [50] between the feature relative importance scores obtained on the considered stage of T2D and those on WT2D (**S2 Table**). The features of T2D_stageII were more correlated to WT2D than were T2D_stageI features, in agreement with the prediction accuracies.

**Fig 7:**
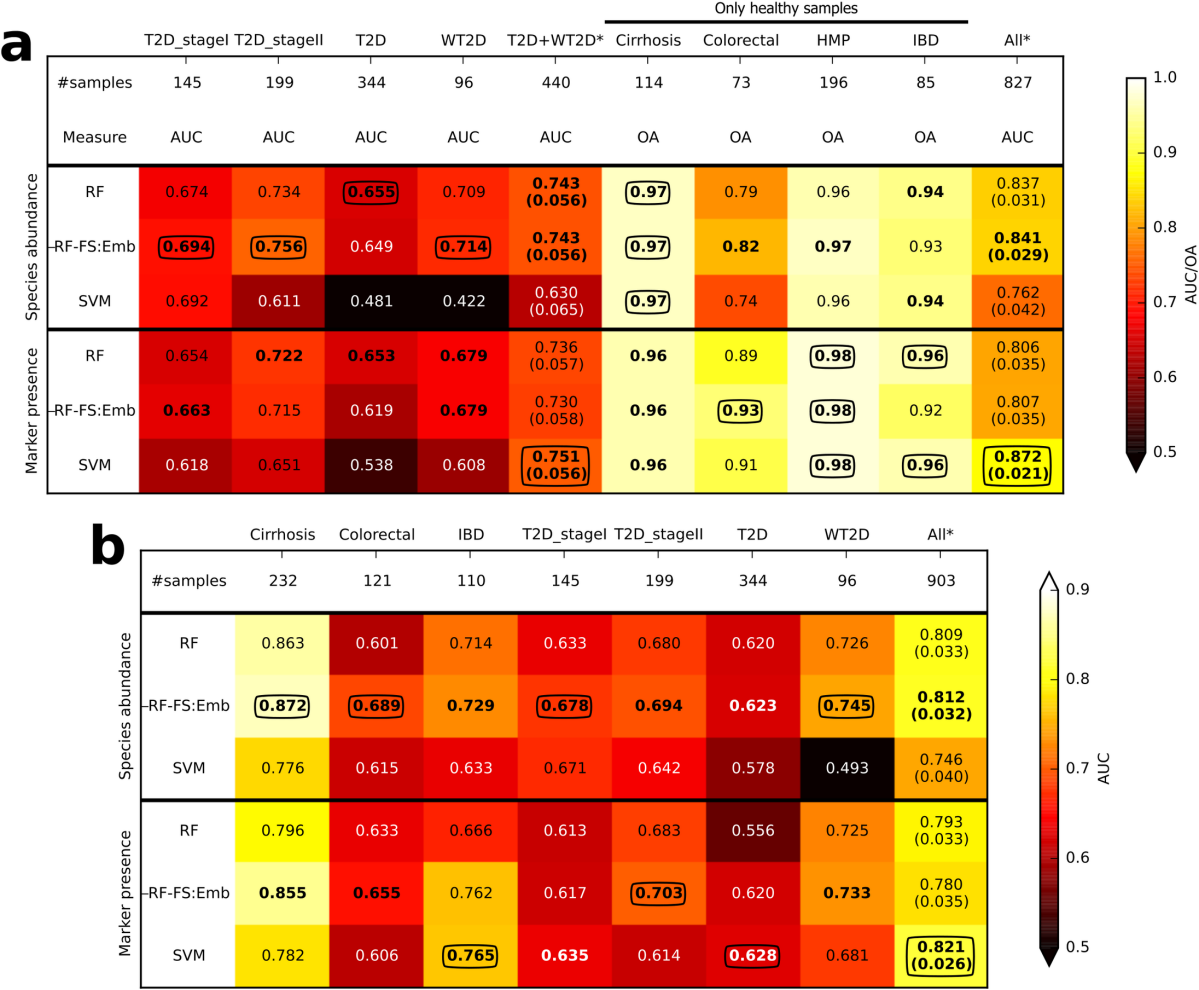
**Cross-study analysis in multiple gut datasets for (a) T2D discrimination and (b) disease discrimination (independently from the type of disease).** For (**a**), we included all the healthy (controls) and diabetes (cases) samples, whereas samples labelled as other diseases were not considered. For (**b**), we instead included all the samples where samples with one of the considered diseases were put together in the same "diseases" class. The * denotes cross-validation results (with the margin of error reported in parenthesis). In the other cases, the model was generated on all the datasets other than the dataset considered for testing, a “leave-one-dataset-out” cross-study validation [51]. For the testing datasets with only healthy samples, prediction accuracy was evaluated in terms of overall accuracy (OA). In bold we report the best value for each scenario and feature type (i.e., species abundance or marker presence), and circled are the absolute best value for each scenario.

Cross-study validation of T2D classification was improved by adding gut microbiome samples from the healthy subjects of four other datasets, i.e., cirrhosis, colorectal, HMP, and IBD, to the training data. While we included all the control groups as “healthy”, there is the potential for health problems among some control subjects. However, it is standard practice in case-control studies to exclude known disease conditions from control groups, so we can assume that, even in the worst case, just a few diseased patients may be included in the controls and these may be mostly due to undiagnosed cases. In this setting we tested the generalization of the model across cohorts (**Fig 7A**) by generating the models on all the available samples apart those associated with the dataset considered for testing, a "leave-one-dataset-out" cross-study validation [51] (denoted as lodoCSV in **Fig 1**). Interestingly, we obtained improved discrimination for T2D when control samples from multiple independent studies were added to training sets, with a high cross-validation AUC score in predicting type-2 diabetes on the entire set of samples (0.837/0.806 for species abundance and marker presence using RF, respectively). These values were in fact higher than the AUC obtained by merging all the T2D and WT2D samples into a single set and cross-validating them (0.743/0.736). This cross-validation accuracy was reduced when we tested the generalization of the model to the two T2D datasets (from 0.743/0.736 to 0.655/0.653 and 0.709/0.679 for T2D and WT2D, respectively), which confirmed a non-complete generalization of the model across cohorts. Interestingly, such values obtained by including healthy samples from other cohorts were again higher than for models constructed only on the T2D or WT2D datasets (**Fig 6**). Thus, including healthy samples from independent cohorts was effective at improving the detection of T2D status. Finally, we evaluated generalization on the healthy samples of the four other datasets (prediction assessed in terms of overall accuracy–OA, right part of **Fig 7A**). In such cases we verified high accuracy (i.e., OA close to 1 for all the considered datasets), confirming correct prediction for most of the control samples. Addition of independent healthy samples to training sets was also performed for gender prediction (**S7 Fig, part b**), also resulting in increased accuracy, although the discrimination capabilities remained generally low. Overall, these results strongly suggest that the inclusion of samples of healthy individuals from unrelated cohorts is beneficial in disease-targeted investigations, especially when the prediction task has to be generalized to new cohorts.

### Avoiding overfitting is crucial to generalization on different cohorts

We compared the cross-validation accuracies that we obtained (**Fig 3**) with results reported in the original papers, when available. For cirrhosis, our best AUC value was 0.963, higher than the cross-validation result reported in [33] (AUC = 0.838). Slight improvements were also verified for colorectal cancer (AUC = 0.881 against the 0.84 reported in [34]). The best AUC for discrimination of IBD patients in the IBD dataset was 0.914, while a similar analysis was not performed in the original paper [35].

For the other datasets (i.e., obesity, T2D, and WT2D), the original works used a two-step procedure that tends to overstate discrimination accuracy: i) first a statistical test was applied on the entire set of samples to select the most discriminative features, then ii) the model was generated on this set of features and the prediction accuracies were estimated directly on the training set or through a cross-validation approach. This approach overestimates accuracy metrics such as AUC because supervised feature selection is applied on the same data used to evaluate the model, a problem referred by the machine learning community as overfitting [52]. When we adopted the same overfitting-prone procedure, our cross-validation accuracy estimates (especially using marker features) were higher than the original ones for all datasets (**S9 Fig)**, but as discussed these are overestimations of the actual discriminative power of the models.

Conversely, the overfitting-prone method resulted in much worse performance when the model was transferred to different cohorts. For example, the results reported in [32] showed an AUC equal to 0.83 when cross-validating on WT2D, which decreased significantly to 0.66 when the model was transferred from the T2D dataset. For the same dataset, we estimated an AUC of 0.785 (**Fig 3**) and 0.701 (**Fig 6**) by (non-overfitted) cross-validation and cross-study validation, respectively. Non-overfitted models in general exhibit cross-validation accuracies that are lower, but better represent the ultimate goal of generalization of the model to independent cohorts.

We stress that the use of a strict, complete cross-validation/cross-study validation approach is necessary in metagenomics. For cross-validation this requires that for each fold, all training steps (including feature selection, model selection, and model construction) are applied on a set of samples that are not overlapping with the samples used for model evaluation/testing. This, together with reducing confounding factors such as antibiotic usage, is necessary for non-overfitted and non-overestimated assessment of the prediction capabilities of metagenomic data.

### Modelling the “healthy” microbiome: Cross-disease prediction

We finally tested the hypothesis that the distinction between the “healthy” and disease-associated gut microbiome can be generalized to diseases for which training information is not available. For this purpose, we considered all gut samples from the disease-associated datasets for a total of 903 samples (**Fig 7B**). Here the class “diseased” included patients affected by the set of disparate diseases discussed above. The cross-validation analysis on the entire set of samples exhibited satisfactory results (AUC = 0.821 for the best model; most discriminative features are reported in **S10 Fig, part a**, although in this scenario the model may in reality classify each type of disease separately from the others. More interesting are the results of cross-study and cross-disease prediction. In such cases the disease associated with the testing cohort was not present in the datasets used to generate the model. Although the obtained AUC were lower than the disease-specific cross-validation results reported previously in **Fig 3**, we still verified in all cases a certain level of generalization of the model. In particular, the AUC varied between 0.628 (for T2D) and 0.872 (for cirrhosis). This represents an intriguing result that can be associated to the task of modelling the features of the “healthy” microbiome for use as a dysbiosis prediction model for syndromes where few or no training samples are available. As expected, several disease-specific species such as *G*. *morbillorum*, *B*. *bifidum* and *P*. *micra* were not among the most discriminative, the diseases with which they are correlated are not in the training set (**S10 Fig, part b**). Conversely, species discriminative for multiple diseases (*S*. *salivarius*, *S*. *anginosus*, *V*. *parvula*, *R*. *intestinalis*, and *C*. *comes*) are even more relevant here, confirming that these species are associated to a general non-healthy microbiome state rather than to specific host conditions (especially *S*. *anginosus*, which is the most relevant feature for four of the five testing datasets). Overall, this suggests that the dysbiosis-associated microbiome is partially distinct from the healthy microbiome regardless of the specific disease under investigation. This also confirms that study-specific confounding factors [44, 45] are only partially affecting the estimation of the classification performance. These species identified as associated to general microbiome dysbiosis should be considered in future microbiome studies as non-specific responses to dysbiosis rather than as organisms directly involved in the pathogenesis of the disease under study.

### Conclusion

We uniformly processed shotgun metagenomic microbiome data for 2424 samples from 8 studies of 6 disease types, and used cross-validation, cross-study validation, and cross-disease validation to evaluate the accuracy of candidate methods of predictive modelling of disease states. We make recommendations of best approaches and non-overfitted practices for using the microbiome as a prediction tool and discuss species and strain-level biomarkers we identified for single and combined datasets. While in this manuscript we focused on taxonomic information, metagenomic functional data such as gene or gene-family abundance data [53] can be exploited in a similar way to conduct a more advanced function-based analysis. Future work will be devoted to exploring more advanced machine learning strategies to further improve classification performance.

In general, cross-validation revealed good prediction capabilities, however classification results varied considerably between prediction tasks. In some cases, the ability to predict disease in undiagnosed cases may be overestimated due to the presence of confounding factors such as active antibiotics treatment. The influence of confounding factors on human microbiome has been scarcely investigated in the literature [54, 55], but recent studies [44, 45] highlight this problem and question the study design of some works.

Cross-study validation involved evaluating the transferability of prediction models between completely independent patient cohorts. We verified generalization across studies, although transferred models were in some cases less accurate than models tested by within-study cross-validation. Interestingly, the inclusion of healthy (control) samples from independent cohorts in training sets was effective for improving the transferability of predictions. We emphasize that considering cross-study performance, instead of the more traditional cross-validation approach, is necessary to understanding prediction capabilities from metagenomic data [56]. Furthermore, avoiding overfitting is crucial for transferring models between different cohorts. Finally, we obtained promising results in the ambitious task of modelling the features of the “healthy” microbiome for use as a dysbiosis prediction model for syndromes where few or no training samples are available. Importantly, this setting is not affected by confounding factors on the target dataset since target samples are not used to build the model. The identified biomarkers for the “healthy” versus “dysbiosis”-associated microbiome are also very important for future microbiome studies of new diseases, because if the same biomarkers are appearing as discriminatory they should be regarded as general dysbiotic organisms rather than microbes directly in involved in the disease under investigation.

Compared to the considerable amount of work done on learning methods for 16S rRNA studies, our contribution emphasized two strengths unique to the shotgun metagenomic approach. First, we showed that improved performance can be achieved using strain-level genomic features (i.e., markers) that are not available from 16S rRNA studies. This is also a confirmation that many disease phenotypes are likely linked with microbial genes and factors that are not “core” components of microbial species, but rather are encoded in variable genomic portions that are strain- or subspecies-specific. Second, despite the common potential biases in DNA extraction, shotgun sequencing is considered more consistent across studies than 16S rRNA sequencing for which different variable regions and primer choices are available [57–59], and thus quantitative microbial signature are inherently less difficult to transfer across cohorts and populations. From a more technical viewpoint, learning analysis in shotgun metagenomes presents distinct challenges due to the very high dimensionality of the dataset when considering strain-level markers (~100K features), requiring different considerations in machine learning than for 16S rRNA datasets. Altogether, we provide the first validated toolbox for disease prediction across studies using shotgun metagenomics.

This study provides a publicly available software framework and uniformly processed microbiome profiles for thousands of samples, to facilitate follow-up studies and evaluation of new methods for classification of disease and other states using metagenomic data. This tool allowed us to assess the predictive power of the microbiome features with respect to disease states and transferability across independent datasets. On a final note, we notice that meta-analyses like the one we performed here were recently regarded as “research parasitism” [60], because we analyse data produced by other laboratories. The analysis of cross-study predictions, and identification of a dysbiotic microbiome, would not be possible any other way. We hope that not only will these results inform future clinical microbiome studies of disease, but that they promote data transparency and re-use as key components of scientific progress.

## Methods

### The proposed tool

We developed a computational tool for metagenomics-based prediction tasks based on machine learning classifiers (i.e., support vector machines (SVMs), random forests (RFs), Lasso, and Elastic Net (ENet)). The tool uses as features quantitative microbiome profiles including species-level relative abundances and presence of strain-specific markers. The framework is fully automatic, including model and feature selection, permitting a systematic and non-overfitted analysis of large metagenomic datasets. Two main kinds of analysis are implemented, i.e., cross-validation (to evaluate the prediction strength of metagenomic data) and cross-study (to evaluate the generalization of the model between different studies). Additionally, the most relevant features are detected for biomarker discovery tasks. Finally, a set of tools is provided to evaluate classification performances in different ways including i) evaluation metrics such as overall accuracy (OA), precision, recall, F1, and area under the curve (AUC); ii) receiver operating characteristic (ROC) curve plots; iii) confusion matrices; iv) plots of the most relevant features in addition to average relative abundances; and v) heatmap figures.

The MetAML (**Met**agenomic prediction **A**nalysis based on **Ma**chine **Le**arning) tool is open-source and available online at http://segatalab.cibio.unitn.it/tools/metaml. All the species-level taxonomic profiling and marker presence and absence data generated by MetaPhlAn2 and used in this paper are available at the same address.

### The adopted machine learning tools

The developed tool incorporates four classification approaches (i.e. SVM, RF, Lasso, and ENet) which have been extensively applied in many different fields including computational biology and genomics [18]. The classifiers were implemented using the scikit-learn python package [61].

SVMs aim at finding the hyperplane that maximizes the margin between the samples in different classes [38], a strategy with many theoretical and practical advantages [62]. Although they are intrinsically linear, they can be extended to the non-linear case by mapping data into a higher dimensional feature space by means of a kernel function. In this work, a radial basis function (RBF) kernel was considered for classifying species abundances, while a linear kernel was adopted for markers due to the sparsity of marker-based profiling. In both cases, the best regularization parameter C (both for linear and RBF kernel) and the width parameter γ (only for RBF kernel) were chosen in {2^−5^, 2^−3^, …, 2^15^} and {2^−15^, 2^−13^, …, 2^3^}, respectively, using a 5-fold stratified cross-validation approach. In cross-validation, samples are first randomly subdivided into *k* subsets (folds) of equal size. In particular, we use here stratified cross-validation, in which folds are made to preserve the percentage of samples of each class. A single subset is then used for the testing the model, and the remaining *k*−1 subsets are used for training. The whole process is repeated *k* times, with each of the *k* subsets used once as the testing set. Finally, the results on the *k* testing folds are averaged to produce a single accuracy evaluation. The parameters that maximize the accuracy (or another metric of choice) are finally chosen. SVMs are binary classifiers and, in this work, extension to multi-class classification problems was obtained through the one-against-one approach [63]. Moreover, class posterior probabilities of each sample were estimated from the predicted labels in the binary case using the Platt formulation [64], which, in the multi-class case, was extended as per [65].

RFs are an ensemble learning method which constructs a large number of decision trees at training time and outputs the class that is the mode of the classes of the individual trees [39]. The free parameters of such classifier were set in this work as follows: i) the number of trees was equal to 500; ii) the number of features to consider when looking for the best split was equal to the root of the number of original features; iii) the quality of a split was measured using the gini impurity criterion. Although a better estimation of such parameters may be obtained through cross-validation, no significant variations were verified by empirical evaluation. We note that RFs can intrinsically deal with binary and multi-class classification problems and give estimation of class probabilities. Moreover, they implicitly provides a list of the features sorted in terms of relative importance. Feature importance was computed in our case using the strategy usually referred to as “gini importance” or “mean decrease impurity” [66]. These importance values were exploited to perform an embedded feature selection strategy (denoted as RF-FS:Emb) implemented as follows: i) RF was applied on the whole set of available features; ii) features were ranked in terms of importance; iii) RF was re-trained on the top *k*-th features, by varying *k* in the set {5, 10, 20, 30, 40, 50, 60, 70, 80, 90, 100, 125, 150, 175, 200}; iv) the number of features that maximized the accuracy was chosen as the optimal number; v) the final model was generated by training RF on this reduced set of features.

Lasso [40] and ENet [41] are generalized linear modelling approaches that incorporate feature selection and regularization to increase prediction accuracy from high-dimensional and collinear predictors. Lasso is based on a multiple logistic regression trained with L1-norm penalized likelihood, while both L1 and L2 norms are penalized in ENet. In this work, we exploited them in two main ways: i) directly applying Lasso or Enet as pure classifiers by training a regression model on the binary classification problem; and ii) using Lasso or ENet for feature selection and then applying SVM or RF on the selected features. In both cases, best regularization parameters were estimated using a 5-fold stratified cross-validation approach. For Lasso this implied to chose the alpha parameter in {10^−4^, …, 10^−0.5^} with values evenly spaced on a logarithmic scale. For ENet, along with alpha the L1_ratio parameter was chosen in [0.1, 0.5, 0.7, 0.9, 0.95, 0.99, 1.0].

### Validation and evaluation strategies

Two main kinds of analysis were performed in this work, i.e., cross-validation and cross-stages/studies. For cross-validation studies, prediction accuracies were assessed by 10-fold cross validation, repeated and averaged on 20 independent runs. We underline that model selection and feature selection are done using only the training set thus avoiding overfitting problems. In the cross-stages/studies case, all the samples of the first stage/study are considered for training and thus used to generate the classification model including the model selection and feature selection steps. The generalization of the model is evaluated by applying it on the samples of the independent stage/study. In all the cases, the results obtained on the original classification problem were compared with those obtained by a random classifier (denoted in the paper as SVM-Shuffled and RF-Shuffled). For such purpose, we applied the same setting after shuffling randomly the labels of all the samples.

Several different metrics were taken into account to evaluate classification performances. First, we considered the OA, which is the percentage of correctly predicted samples. From the confusion matrix three main metrics were computed: i) the precision (i.e., the number of correct positive samples divided by the number of samples predicted as positive); ii) the recall (i.e., the number of correct positive samples divided by the total number of positive samples); iii) the F1 score, which is the harmonic mean of precision and recall, i.e., F1 = 2*(precision*recall)/(precision+recall). These three metrics (which range in [0, 1], where 1 indicates the best case) can be computed for each class separately. For brevity, we report in the paper only the average values: after calculating the metrics for each class, their average values, weighted by the number of samples per class, are computed. For binary classification problems, class posterior probabilities were used to plot the ROC curve, which represents the true positive rate (i.e., the recall) against the false positive rate (i.e., the number of wrong positive samples divided by the total number of non-positive samples). From the ROC curve, we computed the widely-used AUC statistic, which can be interpreted as the probability that the classifier ranks a randomly chosen positive sample higher than a randomly chosen negative one, assuming that positive ranks higher than negative. The AUC ranges in [0.5, 1], where 0.5 corresponds to random change.

In the comparison among classifiers, prediction accuracy was assessed by 10-fold cross-validation, repeated and averaged on 20 independent runs. The same folds were used for all classifiers, i.e. training and test sets were identical for each classifier. In this way, the difference in performance of two classifiers could be calculated directly as the difference in AUCs (or any other metric) within each test fold. Mean difference and standard error were calculated for each 10-fold CV, then averaged across the 20 repetitions for smoothing. 95% confidence intervals on the difference in AUC performance of two classifiers were calculated using the t-distribution with df = 9, i.e.:

$$95\%\;CI:\;\frac{1}{20}\frac{1}{10}\sum_{j=1}^{20}\sum_{i=1}^{10}({AUC}_{1ij}-{AUC}_{2ij})\pm 2.26\times \frac{\sigma_{j}}{\sqrt{10}}$$

where AUC_1ij_ and AUC_2ij_ are the AUC of two classifiers in fold i of repetition j, and σ_j_ is the standard deviation of the AUC_1ij_−AUC_2ij_ across i = 1…10 folds in repetition j. Similarly, *p*-values were obtained from the t-statistic obtained with mean difference and standard error smoothed over the 20 repetitions:

$$t=\frac{\frac{1}{10}\frac{1}{20}\sum_{j=1}^{20}\sum_{i=1}^{10}\left({AUC}_{1ij}-{AUC}_{2ij}\right)}{\frac{1}{20}\sum_{j=1}^{20}\frac{\sigma_{j}}{\sqrt{10}}}$$

using two-tailed t-test with df = 9, noting that the AUC differences were approximately normally distributed.

In terms of feature selection, we reported the list of the 25 most important features found by RFs. For each feature, we considered also the relative importance score, which is a real number in the range [0, 1] with features that sum to 1. Feature selection is done for each run independently, and we report the average results.

### The considered large metagenomic datasets

We initially considered a total of 2571 publicly available metagenomic samples (from eight main studies/datasets) that were reduced to 2424 after pre-processing and curation (see next sections). These are all the human-associated shotgun metagenomic studies with more than 70 samples and read length bigger than 70nt available as of January 2015. Six studies were devoted to the characterization of the human gut microbiome in presence of different diseases. Cirrhosis included 123 patients affected by liver cirrhosis and 114 healthy controls [33]. Colorectal consisted of a total of 156 samples, 53 of which were affected by colorectal cancer [34]. IBD represented the first available large metagenomic dataset and includes 124 individuals, 25 were affected by inflammatory bowel disease (IBD) [35]. Obesity included 123 non-obese and 169 obese individuals [31]. Two distinct studies were instead related to the alteration of the microbiome in subjects with type 2 diabetes (T2D). In the T2D dataset, 170 Chinese T2D patients and 174 non-diabetic controls were present [37]. The WT2D focused on European women and included 53 T2D patients, 49 impaired glucose tolerance individuals and 43 normal glucose tolerance people [32]. Among these six datasets, two of them comprise two independent stages. For cirrhosis, 181 and 56 samples were collected during the so defined discovery and validation phases, respectively. Similarly, for T2D, 145 and 199 samples were acquired during the first (stageI) and second (stageII) stages, respectively. Additionally, two studies focused on healthy subjects and not strictly related to the gut microbiome were also taken into account. HMP included samples collected from five major body sites (i.e., gastrointestinal tract, nasal cavity, oral cavity, skin, and urogenital tract). A subset of these samples were described in [1]. Finally, skin was composed by 291 samples acquired from several different skin sites [36].

### Extraction of species abundance and marker presence profiles from metagenomic samples

The entire analysis was done by taking into account two types of features: species-level relative abundances and presence of strain-specific markers. These features were extracted from the metagenomic samples using MetaPhlAn2 [21] with default parameters. Species abundances are real numbers in the range [0, 1] that sum up to 1 within each sample, while markers assume binary values. Species abundance and marker presence profiles are characterized by very different numbers of features: in the hundreds for species abundance, and hundreds of thousands for markers (the exact numbers of features for each dataset are detailed in **Fig 2**). Before applying MetaPhlAn2 the samples were subject to standard pre-processing as described in the SOP of the Human Microbiome Project [1] without however the step of human DNA removal as these publicly available metagenomes were deposited free from human DNA contamination. Additionally, we removed reads with length less than 90 nucleotides. For the IBD and obesity datasets the minimum length was set to 70 and 75, respectively, as these cohorts were sequenced with shorter read-lengths. Few samples did not pass the minimum length requirement and were thus discarded.

### Experimental setting

The experimental evaluation can be summarized into five main steps: 1) cross-validation analysis was done on the six disease-association datasets for evaluating the capabilities of metagenomic data for disease classification; 2) cross-stage studies were performed on the cirrhosis and T2D datasets in order to test the generalization of the model on independent collection batches from the same study; 3) in terms of T2D, the analysis was extended by taking into account also samples from completely distinct cohorts; 4) cross-studies were also done to model the features of the “healthy” gut microbiome for use as a dysbiosis prediction model for syndromes where few or no training samples are available; 5) cross-validation and cross-study analysis were applied to deal with different classification problem such as gender and body site discrimination. We note that all the investigated classification problems, excluding the body site discrimination, represented binary classification problems. Moreover, most of the analysis was done in terms of disease classification, in which the objective was to discriminate between “healthy” and “diseased” subjects.

1. Cross-validation analysis (see “Cross-validation studies revealed good capabilities for disease prediction”, “Feature selection and strain-specific markers improve prediction accuracy” and "Detection of the disease-associated microbial features") was done on 6 datasets, in which we adopted the same settings used in the original papers. For such reason, in some cases some samples were removed from the analysis. For cirrhosis, all the 232 available samples (subdivided into “healthy” and “affected by liver cirrhosis” subjects) were taken into account. For colorectal we removed the individuals affected by “large adenoma”, which resulted in a total of 121 samples. “Cancer” patients were discriminated from “healthy” subjects, which included also persons affected by “small adenoma”. IBD was composed by a total of 110 samples, in which the “diseased” class included IBD patients affected by both “Crohn's disease” and “ulcerative colitis”. For obesity, we discriminated between “lean” (BMI ≤ 25 kg m ^-2^) and “obese” (BMI ≥ 30 kg m ^-2^) subjects for a total of 253 samples. Individuals having an intermediate BMI (i.e., > 25 and < 30 kg m ^-2^) were excluded. All the 344 samples of the T2D dataset were considered to discriminate between “healthy” individuals and “T2D” patients. The same classification problem was investigated in WT2D, in which 96 samples were taken into account after excluding impaired glucose tolerance women.
2. Cross-stage analysis (see “Metagenomic disease-predictive models show strong cross-stage generalization”) was applied to the cirrhosis and T2D datasets, in which the samples were collected in two independent stages. For cirrhosis, the 232 samples were constituted by 178 samples of discovery and 54 samples of validation. Similarly, for T2D, stageI and stageII included 145 and 199 samples, respectively.
3. Cross-study/cohort analysis was done by taking into account gut samples from multiple datasets. In particular, we focused on the discrimination between “healthy” and “T2D” subjects (see “Cross-study generalization is improved by including healthy samples from other cohorts”). For this purpose, in addition to the T2D and WT2D datasets, we considered also the “healthy” samples from four other datasets, for which “diseased” samples were excluded since not related to “T2D”: cirrhosis, colorectal, HMP and IBD for a total of 908 samples. The entire analysis (apart for the two experiments denoted in the figure with the *) was done to evaluate the generalization of the model, therefore all the samples were used for training apart the dataset considered for test. For comparison, we investigated also cross-validation studies (denoted with the *). In such case, we considered a single sample per subject in order to avoid overestimation of the accuracies (this issue was present in the HMP dataset only). The sample was chosen randomly from the available samples for each iteration. So the cross-validation on the entire set of samples (denoted as All*) was done on a total of 827 samples.
4. Cross-study analysis was also applied to discriminate between “healthy” and “diseased” subjects (independently from the type of disease) in gut samples (see “Modelling the “healthy” microbiome: cross-disease prediction”). We considered five different datasets (i.e., cirrhosis, colorectal, IBD, T2D, and WT2D) for a total of 903 samples. Therefore the class “diseased” included patients affected by liver cirrhosis, colorectal cancer, IBD and T2D. Also in this case all the analysis was done to evaluate model generalization, apart the cross-validation study on the entire set of samples (denoted as All*).
5. Finally, the developed tool was applied to non-disease-based classification problems (see “Extension to non-disease classification problems” and “Cross-study generalization is improved by including healthy samples from other cohorts”). First, we considered gender classification, in which therefore “female” were discriminated from “male” subjects. In this case, we took into account six datasets acquired from different body sites: four from gut (i.e., cirrhosis, colorectal, IBD, and T2D), one from skin (i.e., skin), and one from multiple sites (i.e., HMP). First, we did cross-validation studies, in which each dataset was analyzed independently from the others. Also in this case we considered only one sample per subject in order to avoid overestimation of the accuracies. For such reason, HMP and skin were reduced to 131 and 17 samples, respectively. Then, we performed a cross-study analysis on gut samples (thus the entire skin and part of the HMP datasets were excluded). In such case, we considered first all the available samples (for a total of 1016 samples) and then only the “healthy” samples (for a total of 642 samples). Then, the implemented tool was validated on a multi-class classification problem, which was represented by body site discrimination in the HMP dataset. The available samples were subdivided into five major body sites [1]: gastrointestinal tract, nasal cavity, oral cavity, skin, and urogenital tract. Cross-validation analysis was done by considering one sample per subject. Moreover, we considered also the case in which more than one sample per subject, but each from a different body site, was taken into account.

### Code and data availability

The MetAML (**Met**agenomic prediction **A**nalysis based on **M**achine **L**earning) software is open-source, written in Python and available online at http://segatalab.cibio.unitn.it/tools/metaml together with all the data used and discussed in this work.

## Supporting Information

S1 Table Statistical test against the null hypothesis of equal AUC for classification of true and shuffled labels using RF and SVM. The table reports the *p*-value for cross-validation analysis for disease discrimination on six different datasets using species abundance as microbiome features. Average values with margins of error for AUC are reported in **Fig 1**.
(PDF)

S2 Table Cross-study analysis for T2D discrimination in the T2D and WT2D datasets using RF on species abundances. The AUC is computed by training the model on one stage of T2D and testing it on WT2D (same results in **Fig 5**). The IntCorr is the integrative correlation [50] between the feature relative importance scores obtained on the considered stage of T2D and those on WT2D.
(PDF)

S1 Fig Statistical test to compare classification of true labels using RF and SVM. The table reports the *p*-values for the null hypothesis of no difference in AUC by 10-fold cross-validation, assessed by paired t-test, for disease discrimination on six different datasets using species abundance and marker presence as microbiome features. Comparisons that are statistically different (*p*-value < 0.05) are highlighted in red. Average values with margins of error for AUC are reported in **Fig 2**.
(TIFF)

S2 Fig Average number of features selected by the different methods for disease discrimination in different cross-validation studies. The corresponding prediction performances are reported in **Fig 3** and **S3 Fig**.
(TIF)

S3 Fig Prediction performances (assessed using AUC) for disease discrimination in different cross-validation studies using Elastic Net (Enet) and Lasso. Species abundance and marker presence were used as microbiome features. The best value for each dataset and feature type (i.e., species abundance or marker presence) are in bold, and the overall best values for each dataset are circled. Enet and Lasso denote their use as classifier applied on the entire set of features; RF(or SVM)-FS:Enet(or Lasso) denotes RF (or SVM) used as classifier on the reduced set of features given by Enet (or Lasso). The margins of error are reported in parenthesis.
(TIF)

S4 Fig **Most important discriminating species (left) and markers (right) identified by RF for disease discrimination in (a) IBD, (b) obesity, (c) T2D and (d) WT2D cross-validation studies.** In the left panels, for each species reported on the vertical axis, the top bar (in blue) corresponds to the feature relative importance (with standard deviation reported with error bars) and the two bottom bars refer to the average relative abundance for healthy (in green) and diseased (in red) samples. In the right panels, for each marker the top bar is coloured according to the corresponding species and the two bottom bars refer to the average marker presence.
(TIF)

S5 Fig Correlation between feature relative importance determined by RF and species relative abundance in six different cross-validation studies for disease discrimination. (TIF)

S6 Fig Hierarchical clustering on the feature relative importance score determined by running RF on species abundances in six different cross-validation studies for disease discrimination. Features and data sets were clustered using correlation similarity.
(TIF)

S7 Fig **(a) Cross-validation and (b) cross-study analysis for gender discrimination in multiple datasets**. In bold: the best value for each scenario and feature type (i.e., species abundance or marker presence); Circled: the absolute best value for each scenario.
(TIF)

S8 Fig Body site discrimination for the HMP dataset. (**a**) Cross-validation results by considering one sample per subject or more than one sample per subject but each from a different body site. (**b**) Normalized confusion matrix when using RF on species abundance.
(TIF)

S9 Fig The importance of cross-validation analysis. Prediction capabilities for disease discrimination in the (**a**) obesity, (**b**) T2D and (**c**) WT2D datasets are overestimated if the most relevant features (i.e., species abundance or marker presence) are pre-selected. Cross-validation prevents this problem.
(TIF)

S10 Fig Most relevant features to discriminate between “healthy” and “diseased” subjects independently from the type of disease in multiple gut datasets. (**a**) Most relevant species (left) and markers (right) identified by RF by cross-validating on the entire set of samples. In the left panel, for each species reported on the vertical axis, the top bar (in blue) corresponds to the feature relative importance (with standard deviation reported with error bars) and the two bottom bars refer to the average relative abundance for healthy (in green) and diseased (in red) samples. In the right panel, for each marker the top bar is coloured according to the corresponding species and the two bottom bars refer to the average marker presence. (**b**) Hierarchical clustering on the feature relative importance score determined by running RF on species abundances in a "leave-one-dataset-out" cross-study validation [51]. For each case the model was generated on all the datasets other than the dataset considered for testing. Features and data sets were clustered using correlation similarity.
(TIF)
