# Supplementary material for: Machine Learning Meta-analysis of Large Metagenomic Datasets: Tools and Biological Insights
Source: PLoS Comput Biol. 2016 Jul 11;12(7):e1004977. doi: 10.1371/journal.pcbi.1004977 (PMC4939962; doi:10.1371/journal.pcbi.1004977)
Supplement: S1 Table — The table reports the p-value for cross-validation analysis for disease discrimination on six different datasets using species abundance as microbiome features. Average values with margins of error for AUC are reported in Fig 1. (PDF) [file pcbi.1004977.s001.pdf]

	Cirrhosis	Colorectal	IBD	Obesity	T2D	WT2D
RF	6.4×10^{-7}	5.0×10^{-5}	4.0×10^{-4}	9.9×10^{-3}	7.6×10^{-5}	4.5×10^{-3}
SVM	5.6×10^{-7}	9.4×10^{-4}	1.9×10^{-4}	1.3×10^{-3}	6.4×10^{-4}	7.5×10^{-3}
