# Supplementary material for: Machine Learning Meta-analysis of Large Metagenomic Datasets: Tools and Biological Insights
Source: PLoS Comput Biol. 2016 Jul 11;12(7):e1004977. doi: 10.1371/journal.pcbi.1004977 (PMC4939962; doi:10.1371/journal.pcbi.1004977)
Supplement: S2 Table — The AUC is computed by training the model on one stage of T2D and testing it on WT2D (same results in Fig 5). The IntCorr is the integrative correlation [50] between the feature relative importance scores obtained on the considered stage of T2D and those on WT2D. (PDF) [file pcbi.1004977.s002.pdf]

Training on	AUC on WT2D	IntCorr
T2D_stageI	0.585	0.575
T2D_stageII	0.689	0.624
T2D	0.664	0.628
