# Supplementary figures and images for: Machine Learning Meta-analysis of Large Metagenomic Datasets: Tools and Biological Insights
Source: PLoS Comput Biol. 2016 Jul 11;12(7):e1004977. doi: 10.1371/journal.pcbi.1004977 (PMC4939962; doi:10.1371/journal.pcbi.1004977)

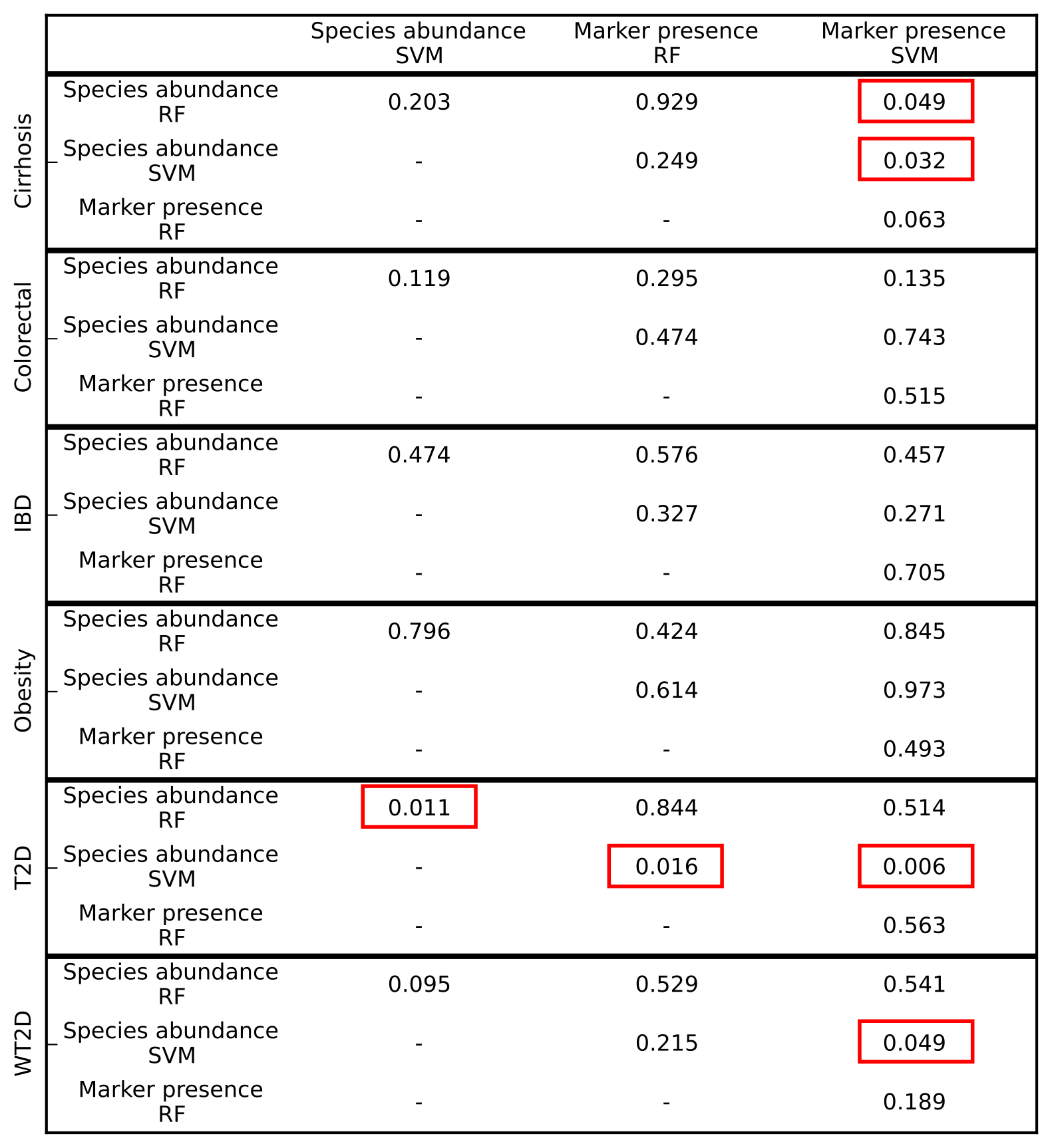

Supplement: S1 Fig — The table reports the p-values for the null hypothesis of no difference in AUC by 10-fold cross-validation, assessed by paired t-test, for disease discrimination on six different datasets using species abundance and marker presence as microbiome features. Comparisons that are statistically different (p-value < 0.05) are highlighted in red. Average values with margins of error for AUC are reported in Fig 2. (TIFF) [file pcbi.1004977.s003.tiff]

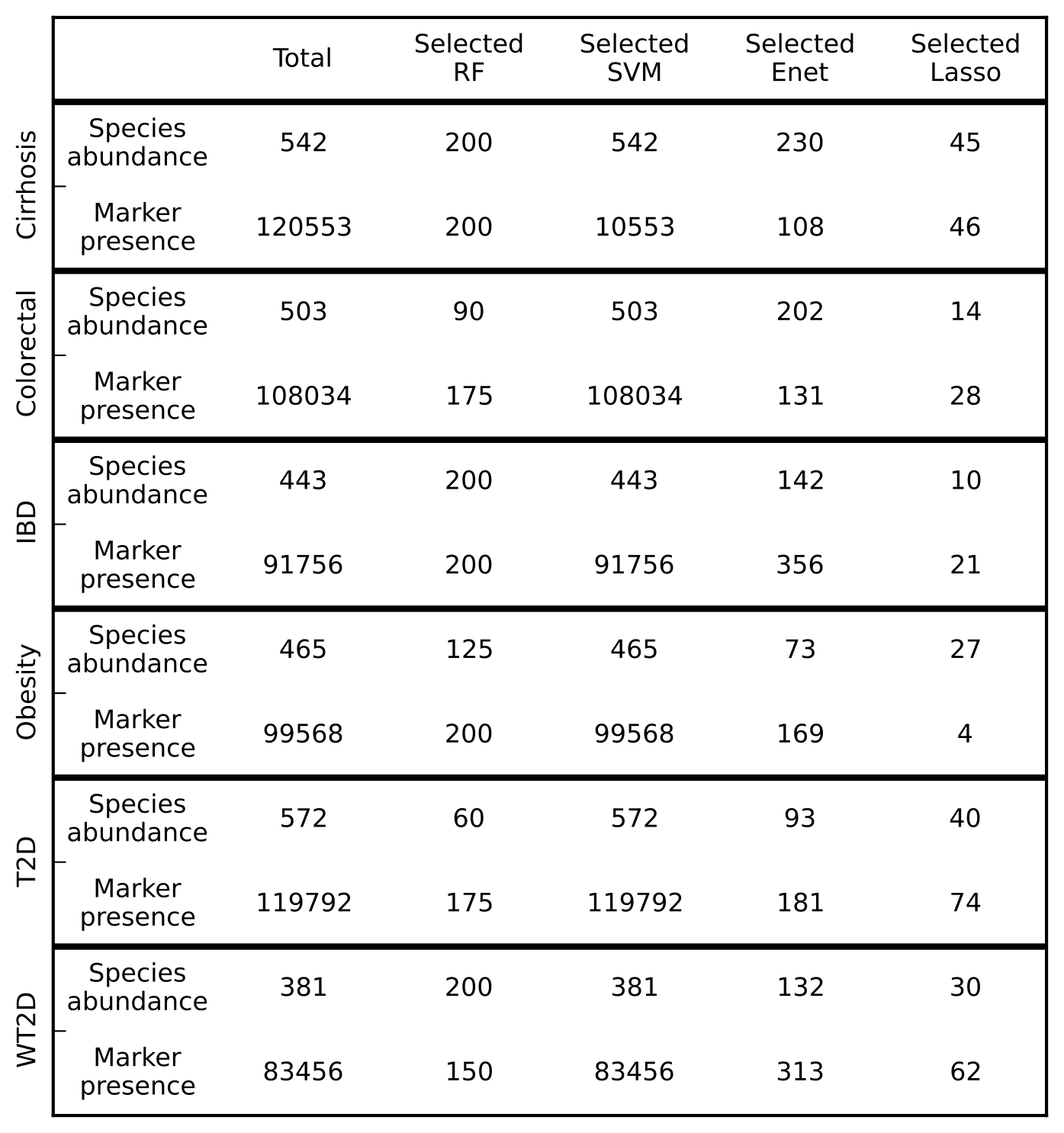

Supplement: S2 Fig — The corresponding prediction performances are reported in Fig 3 and S3 Fig. (TIF) [file pcbi.1004977.s004.tif]

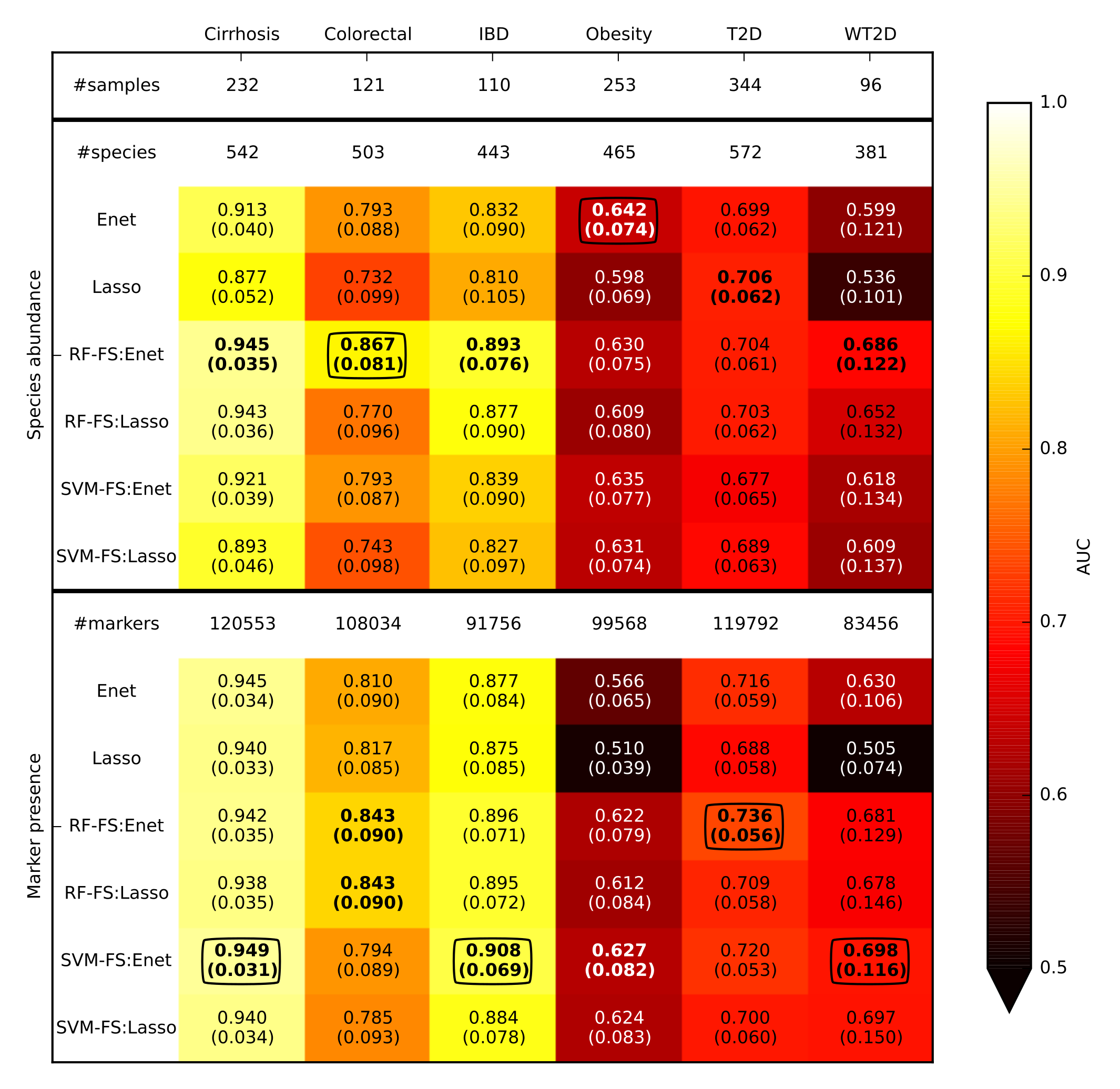

Supplement: S3 Fig — Species abundance and marker presence were used as microbiome features. The best value for each dataset and feature type (i.e., species abundance or marker presence) are in bold, and the overall best values for each dataset are circled. Enet and Lasso denote their use as classifier applied on the entire set of features; RF(or SVM)-FS:Enet(or Lasso) denotes RF (or SVM) used as classifier on the reduced set of features given by Enet (or Lasso). The margins of error are reported in parenthesis. (TIF) [file pcbi.1004977.s005.tif]

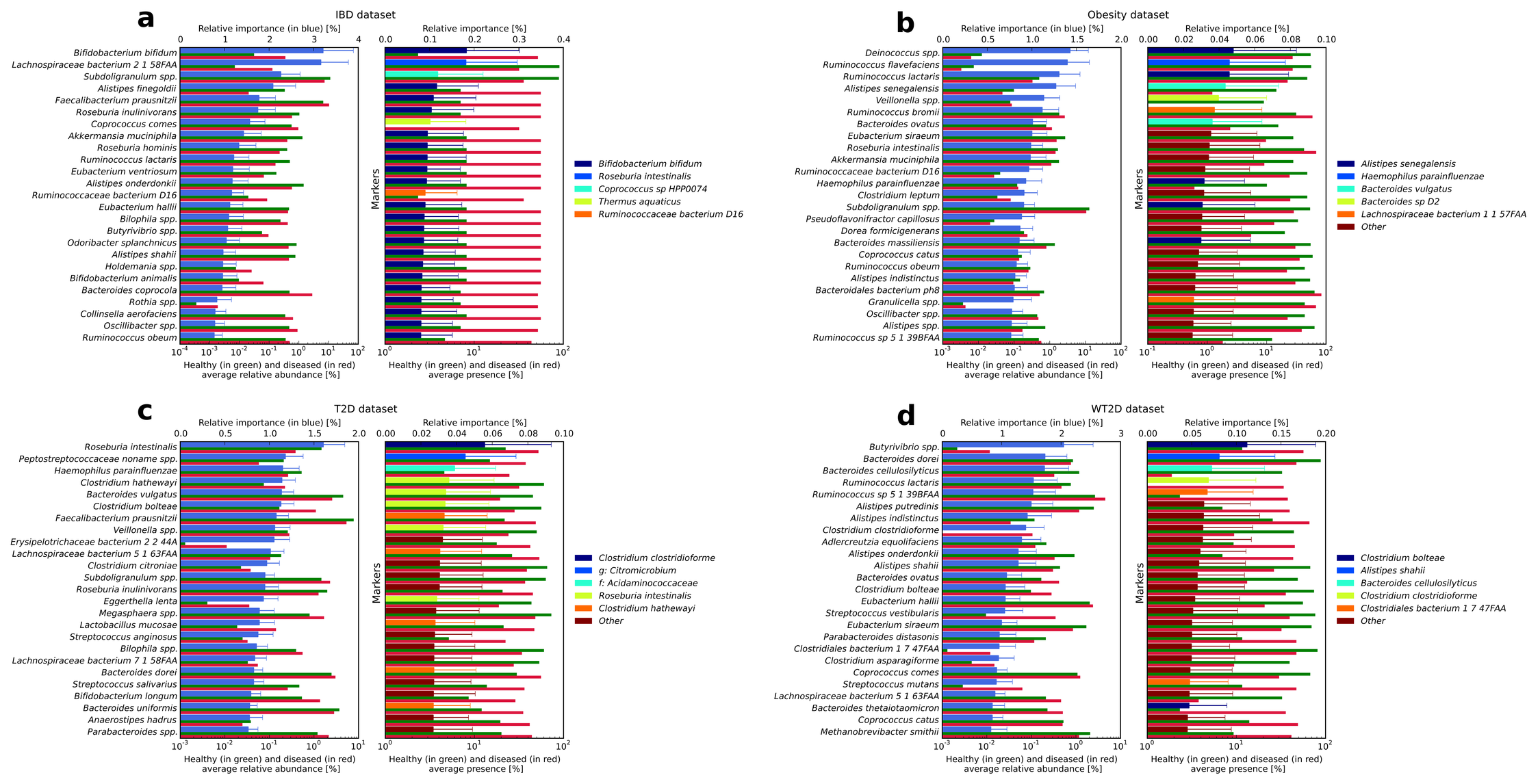

Supplement: S4 Fig — Most important discriminating species (left) and markers (right) identified by RF for disease discrimination in (a) IBD, (b) obesity, (c) T2D and (d) WT2D cross-validation studies. In the left panels, for each species reported on the vertical axis, the top bar (in blue) corresponds to the feature relative importance (with standard deviation reported with error bars) and the two bottom bars refer to the average relative abundance for healthy (in green) and diseased (in red) samples. In the right panels, for each marker the top bar is coloured according to the corresponding species and the two bottom bars refer to the average marker presence. (TIF) [file pcbi.1004977.s006.tif]

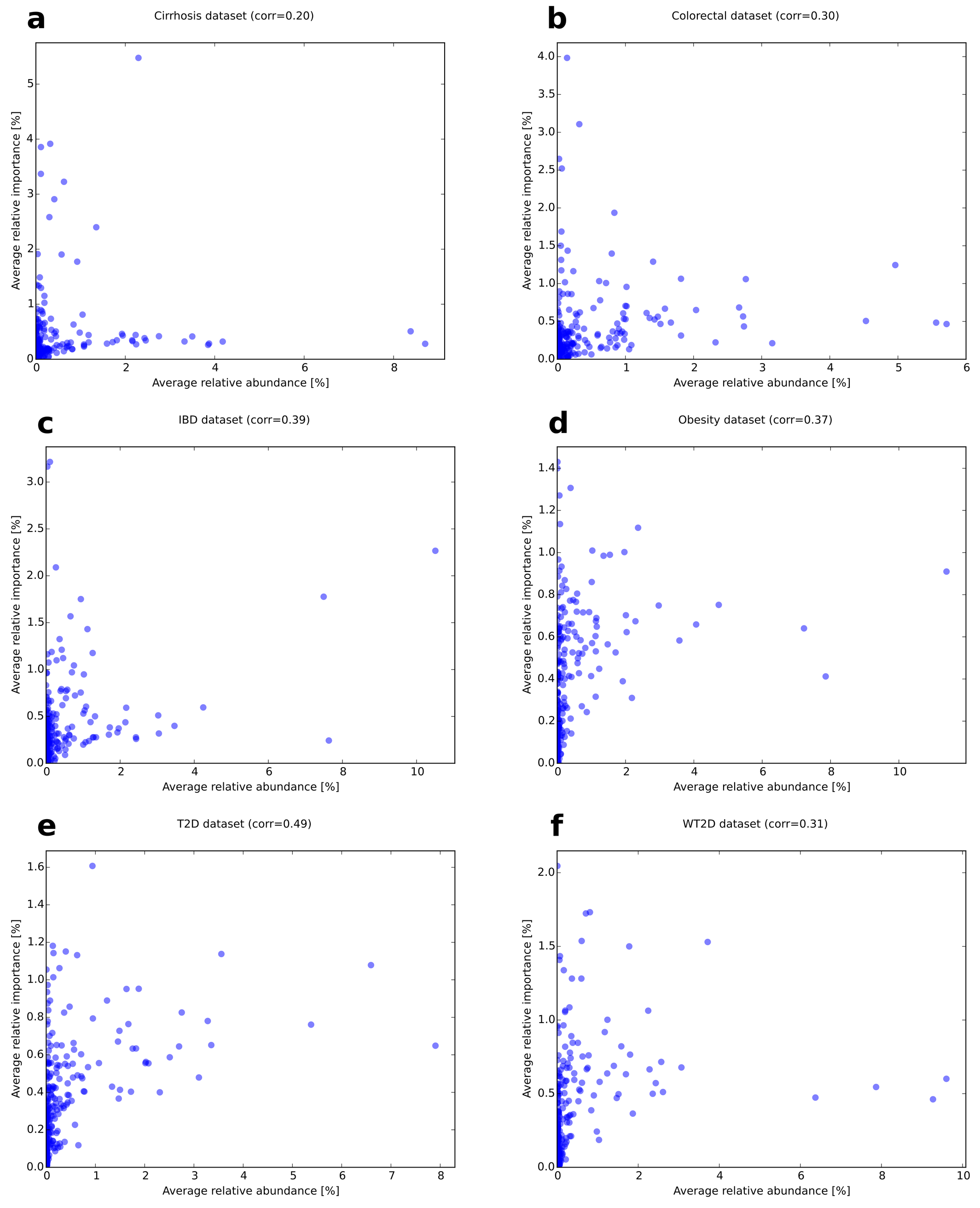

Supplement: S5 Fig — (TIF) [file pcbi.1004977.s007.tif]

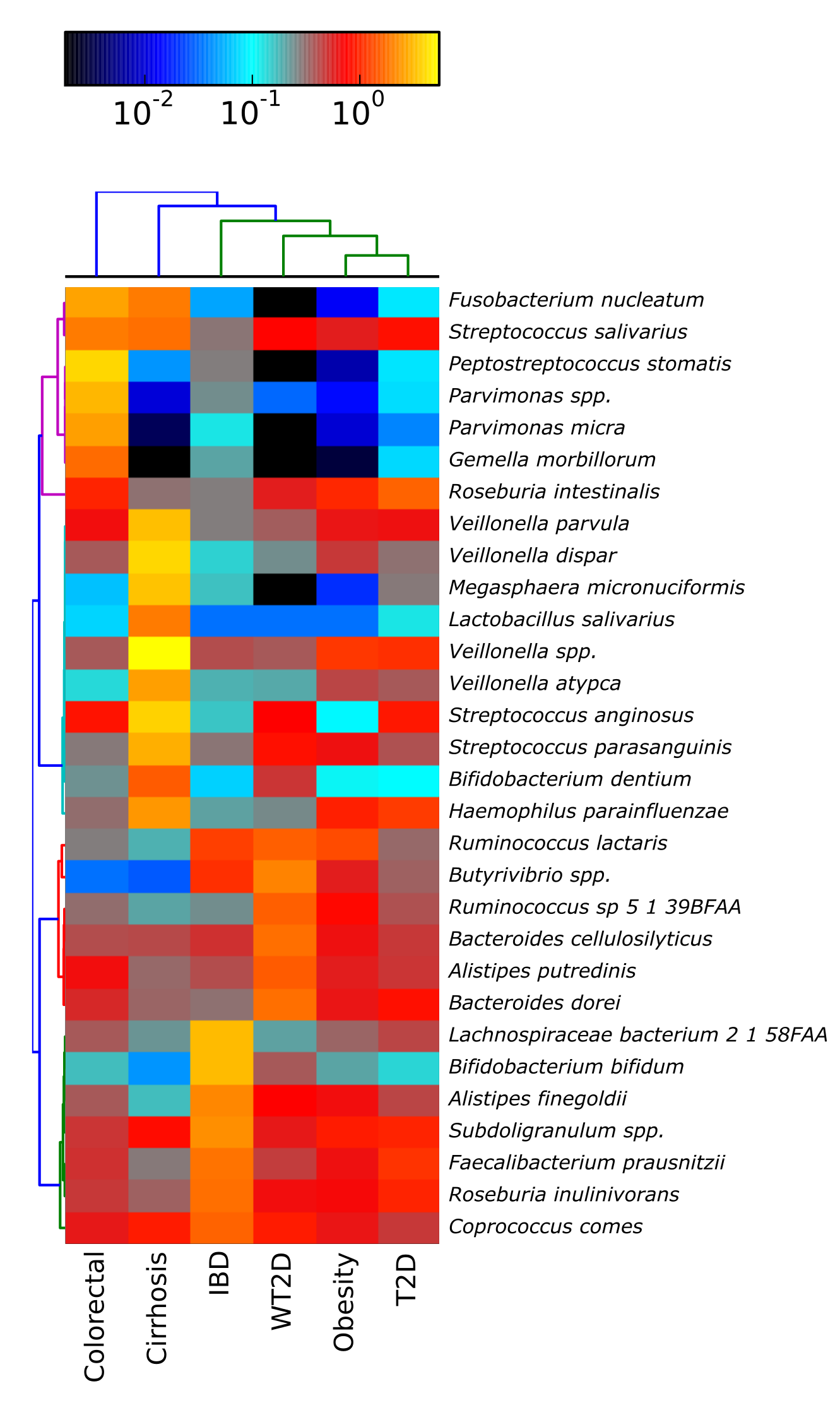

Supplement: S6 Fig — Features and data sets were clustered using correlation similarity. (TIF) [file pcbi.1004977.s008.tif]

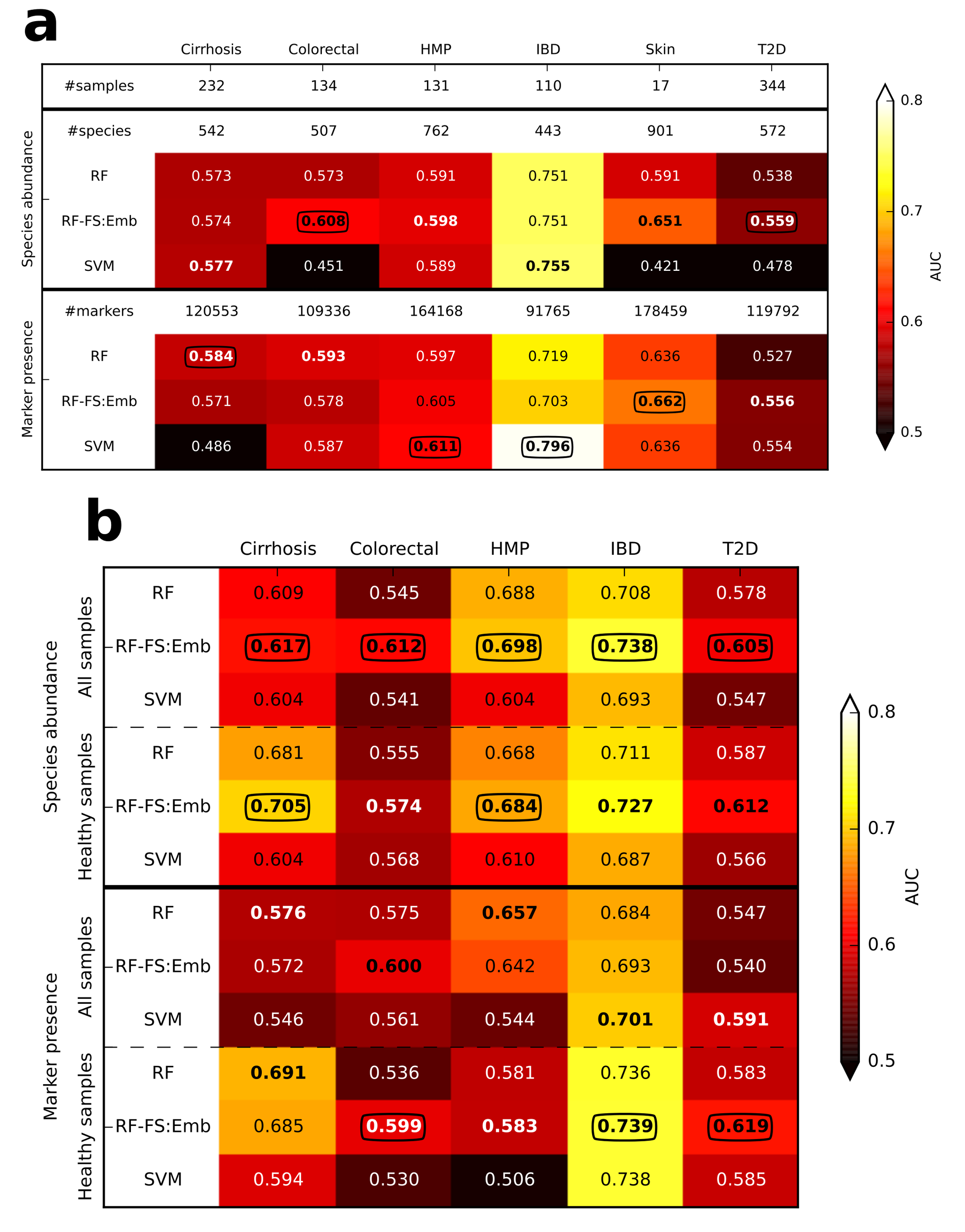

Supplement: S7 Fig — (a) Cross-validation and (b) cross-study analysis for gender discrimination in multiple datasets. In bold: the best value for each scenario and feature type (i.e., species abundance or marker presence); Circled: the absolute best value for each scenario. (TIF) [file pcbi.1004977.s009.tif]

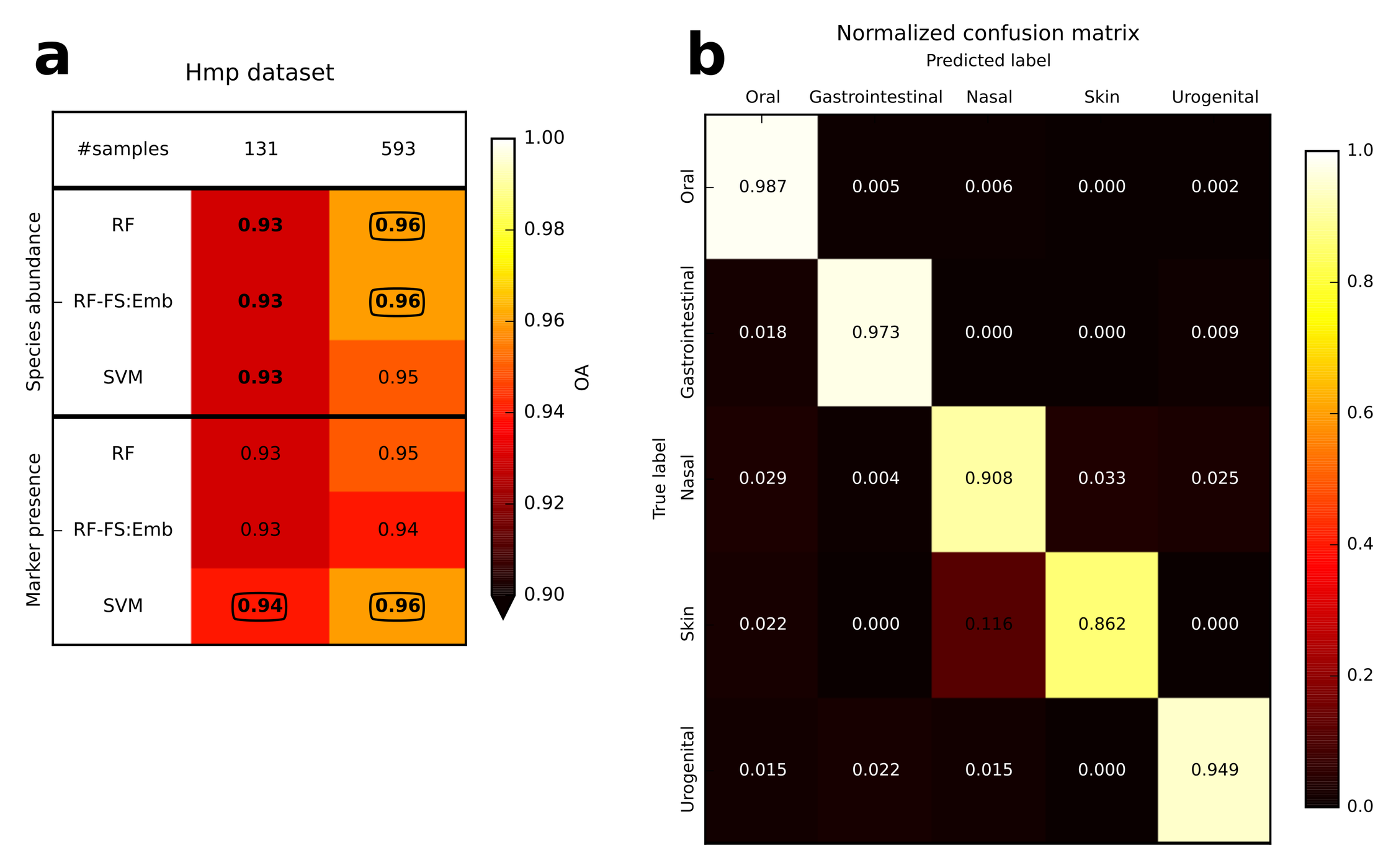

Supplement: S8 Fig — (a) Cross-validation results by considering one sample per subject or more than one sample per subject but each from a different body site. (b) Normalized confusion matrix when using RF on species abundance. (TIF) [file pcbi.1004977.s010.tif]

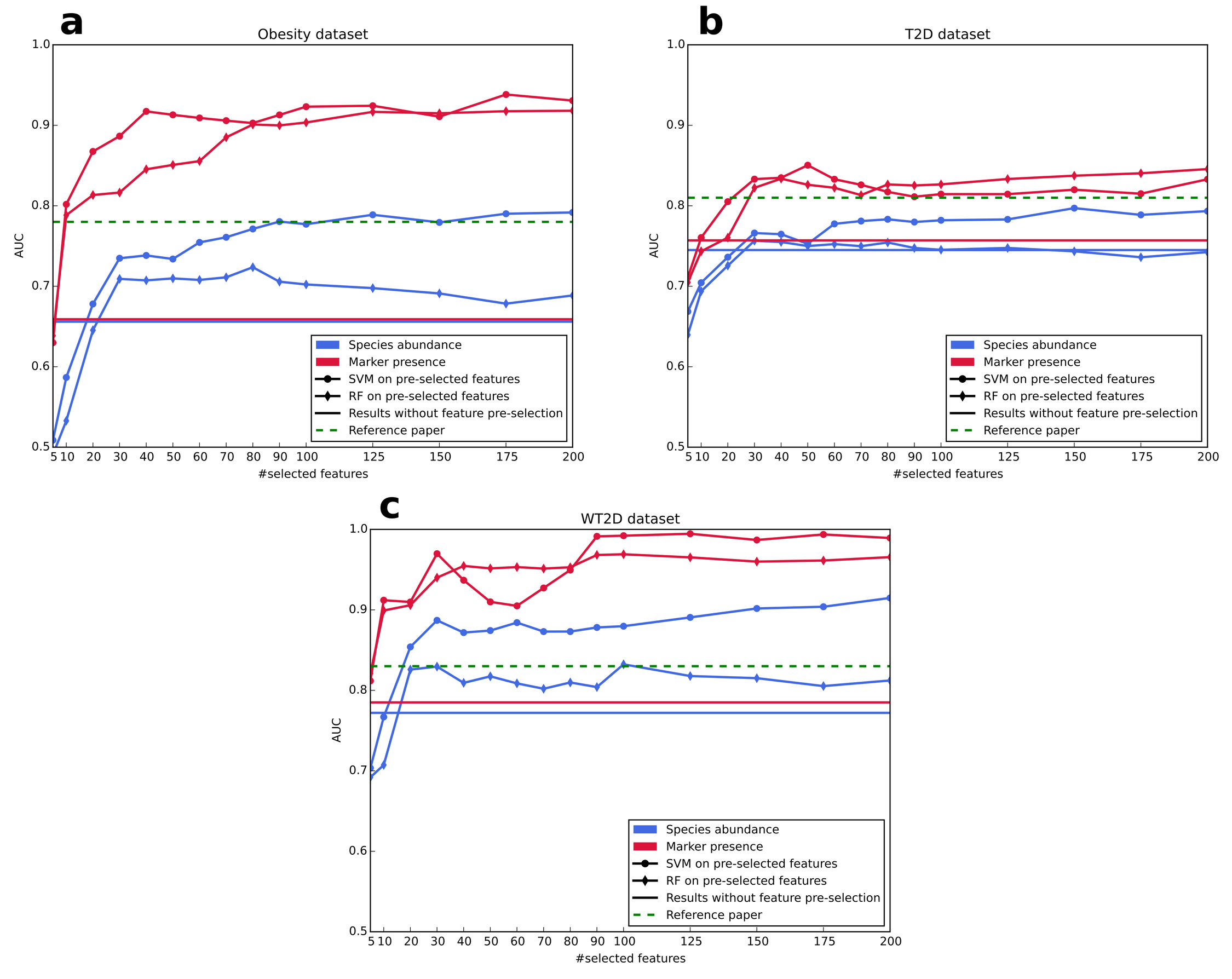

Supplement: S9 Fig — Prediction capabilities for disease discrimination in the (a) obesity, (b) T2D and (c) WT2D datasets are overestimated if the most relevant features (i.e., species abundance or marker presence) are pre-selected. Cross-validation prevents this problem. (TIF) [file pcbi.1004977.s011.tif]

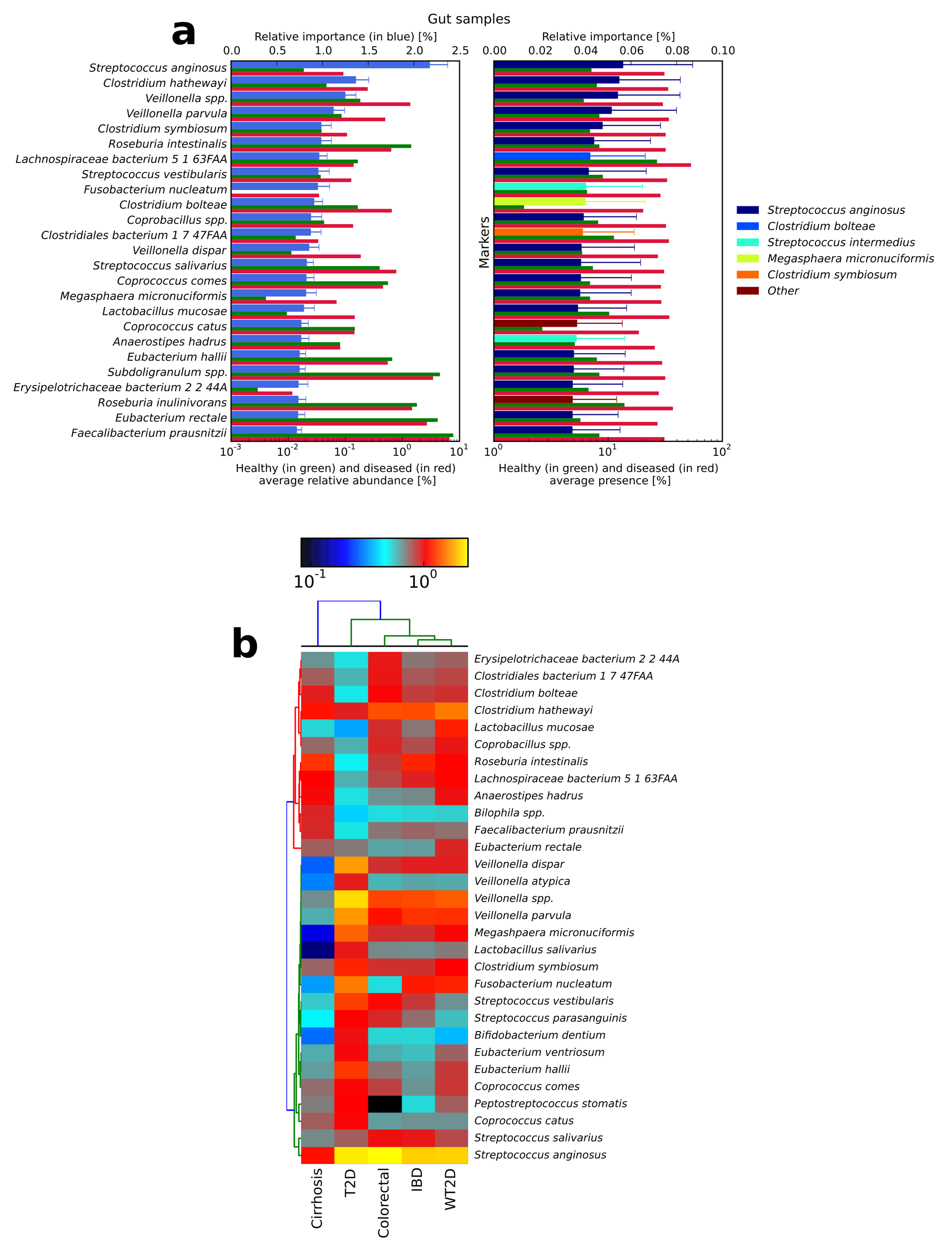

Supplement: S10 Fig — (a) Most relevant species (left) and markers (right) identified by RF by cross-validating on the entire set of samples. In the left panel, for each species reported on the vertical axis, the top bar (in blue) corresponds to the feature relative importance (with standard deviation reported with error bars) and the two bottom bars refer to the average relative abundance for healthy (in green) and diseased (in red) samples. In the right panel, for each marker the top bar is coloured according to the corresponding species and the two bottom bars refer to the average marker presence. (b) Hierarchical clustering on the feature relative importance score determined by running RF on species abundances in a "leave-one-dataset-out" cross-study validation [51]. For each case the model was generated on all the datasets other than the dataset considered for testing. Features and data sets were clustered using correlation similarity. (TIF) [file pcbi.1004977.s012.tif]
